# A transcriptional cross species map of pancreatic islet cells

**DOI:** 10.1016/j.molmet.2022.101595

**Published:** 2022-09-13

**Authors:** Sophie Tritschler, Moritz Thomas, Anika Böttcher, Barbara Ludwig, Janine Schmid, Undine Schubert, Elisabeth Kemter, Eckhard Wolf, Heiko Lickert, Fabian J. Theis

**Affiliations:** 1 Institute of Computational Biology, Helmholtz Zentrum München, 85764 Neuherberg, Germany; 2 Institute of Diabetes and Regeneration Research, Helmholtz Zentrum München, 85764 Neuherberg, Germany; 3 Technical University of Munich, School of Life Sciences Weihenstephan, 85354 Freising, Germany; 4 Institute of AI for Health, Helmholtz Zentrum München, 85764 Neuherberg, Germany; 5 Institute of Stem Cell Research, Helmholtz Zentrum München, 85764 Neuherberg, Germany; 6 Department of Medicine III, University Hospital Carl Gustav Carus, Technical University of Dresden, 01307 Dresden, Germany; 7 Paul Langerhans Institute Dresden of Helmholtz Zentrum München, University Hospital Carl Gustav Carus, Technical University of Dresden, 01307 Dresden, Germany; 8 German Center for Diabetes Research (DZD), 85764 Neuherberg, Germany; 9 Chair for Molecular Animal Breeding and Biotechnology, Gene Center, LMU Munich, 81377 Munich, Germany; 10 Center for Innovative Medical Models (CiMM), Department of Veterinary Sciences, LMU Munich, 85764 Oberschleißheim, Germany; 11 Technical University of Munich, Medical Faculty, 81675 Munich, Germany; 12 Technical University of Munich, Department of Mathematics, 85748 Garching b. Munich, Germany

**Keywords:** Pancreatic islets, β-Cell, α-Cell, Single-cell RNAseq, Cross species conservation, Translation

## Abstract

**Objective:**

Pancreatic islets of Langerhans secrete hormones to regulate systemic glucose levels. Emerging evidence suggests that islet cells are functionally heterogeneous to allow a fine-tuned and efficient endocrine response to physiological changes. A precise description of the molecular basis of this heterogeneity, in particular linking animal models to human islets, is an important step towards identifying the factors critical for endocrine cell function in physiological and pathophysiological conditions.

**Methods:**

In this study, we used single-cell RNA sequencing to profile more than 50′000 endocrine cells isolated from healthy human, pig and mouse pancreatic islets and characterize transcriptional heterogeneity and evolutionary conservation of those cells across the three species. We systematically delineated endocrine cell types and α- and β-cell heterogeneity through prior knowledge- and data-driven gene sets shared across species, which altogether capture common and differential cellular properties, transcriptional dynamics and putative driving factors of state transitions.

**Results:**

We showed that global endocrine expression profiles correlate, and that critical identity and functional markers are shared between species, while only approximately 20% of cell type enriched expression is conserved. We resolved distinct human α- and β-cell states that form continuous transcriptional landscapes. These states differentially activate maturation and hormone secretion programs, which are related to regulatory hormone receptor expression, signaling pathways and different types of cellular stress responses. Finally, we mapped mouse and pig cells to the human reference and observed that the spectrum of human α- and β-cell heterogeneity and aspects of such functional gene expression are better recapitulated in the pig than mouse data.

**Conclusions:**

Here, we provide a high-resolution transcriptional map of healthy human islet cells and their murine and porcine counterparts, which is easily queryable via an online interface. This comprehensive resource informs future efforts that focus on pancreatic endocrine function, failure and regeneration, and enables to assess molecular conservation in islet biology across species for translational purposes.

## Highlights

• We provide queryable transcriptional maps of >50′000 pancreatic islet cells from human, pig and mouse.
• This resource enables studying islet cell heterogeneity, function, maturation and stress across species.
• We describe human α- and β-cell states that activate distinct programs in response to external stressors.
• Multiple functional and regulatory human expression units are better mirrored in pigs than mice.
• Heterogeneity in humans related to stress is better conserved in pig than in mouse cells.

## 1 Introduction

Pancreatic β-cells are essential endocrine cells, which regulate systemic glucose homeostasis together with the other endocrine islet cells - glucagon-producing α-cells, somatostatin-producing δ-cells, pancreatic polypeptide-producing PP-cells and ghrelin-producing ε-cells. In diabetic patients, β-cells are lost or become dysfunctional, which leads to chronically elevated blood glucose levels. Even in healthy individuals, β-cells are heterogeneous and differ in their responsiveness to glucose, insulin secretion capacity, maturation state, stress response and other functional phenotypes [[1], [2], [3]]. Similarly, varying phenotypes and cell states of α-cells have been described [[4], [5], [6]]. It has been proposed that these molecular and functional cell states complement each other to fine tune and efficiently adapt the endocrine response to physiological changes in their environment [3,7,8]. Heterogeneity can also arise from individual cells that cycle asynchronously between phases of active insulin biosynthesis, recovery and rest [9], different tissue locations or phenotypic variation between cells of different ages [10]. Although it is unclear to which extent the endocrine heterogeneity is important for normal pancreatic endocrine function, a precise description of the functional and molecular differences between distinct cell states informs drug discovery and development of anti-diabetic drugs [4,[11], [12], [13], [14]]. Most importantly, it will help to establish a reference for a mature, functional β-cell as a clinical endpoint. Moreover, aspects of the molecular programs that characterize less-functional or stressed states, may overlap with programs that contribute to pathological β-cell dysfunction in diabetes and thus reveal novel molecular targets. Lastly, it can indicate which subset of cells has the potential to respond to a treatment, which affects the efficacy of a therapeutic approach.

Today, most of the pre-clinical research of the endocrine system relies on animal models as access to pancreatic tissue from patients is limited. Endocrine cells are mostly studied in rodents. However, differences in endocrine development and whole-body anatomy and physiology between human and rodents lowers the predictive value of rodent models for human physiology and therapeutic success [15]. As an alternative to rodents, pigs are a large-animal model with a higher translational promise: The anatomy and physiology of pigs is more similar to humans, porcine islets are a potential source for islet xenotransplantation, and, ethical concerns about animal studies are smaller for pigs than for non-human primates [[16], [17], [18], [19]]. Still, it is unclear whether human functional states and molecular profiles of endocrine cells are better conserved in pigs than rodents [20].

Only recently, endocrine heterogeneity can be systematically characterized at the molecular level by profiling individual cells with high-throughput single-cell RNA sequencing [12]. Most phenotypic states are reflected in the gene or protein expression profile of a cell and can thus be captured and resolved by single-cell approaches. Single-cell studies have provided cell-by-cell descriptions of healthy and diabetic pancreatic islets from mice [11,21,22] and human donors [4,9,13,14,[23], [24], [25]], however in these early studies resolution was limited by low cell numbers - which makes it difficult to identify rare cell states and to infer cell state transitions - and there is so far no systematic cross-species comparison. Here, we leveraged single-cell transcriptomics to finely resolve human endocrine heterogeneity and its conservation in pig and mouse islets. We describe endocrine cell type signatures and gradients as well as distinct α- and β-cell states that can be related to distinct biological properties like function, maturation and cellular stress. Our data represents a queryable resource to provide insight into shared endocrine cell states and expression profiles in humans, pigs and mice, which can be easily accessed and explored online and adheres to the FAIR data guiding principles [26].

## 2 Results

### 2.1 Conservation of endocrine signatures across human, pig, and mouse

**Figure 1:**
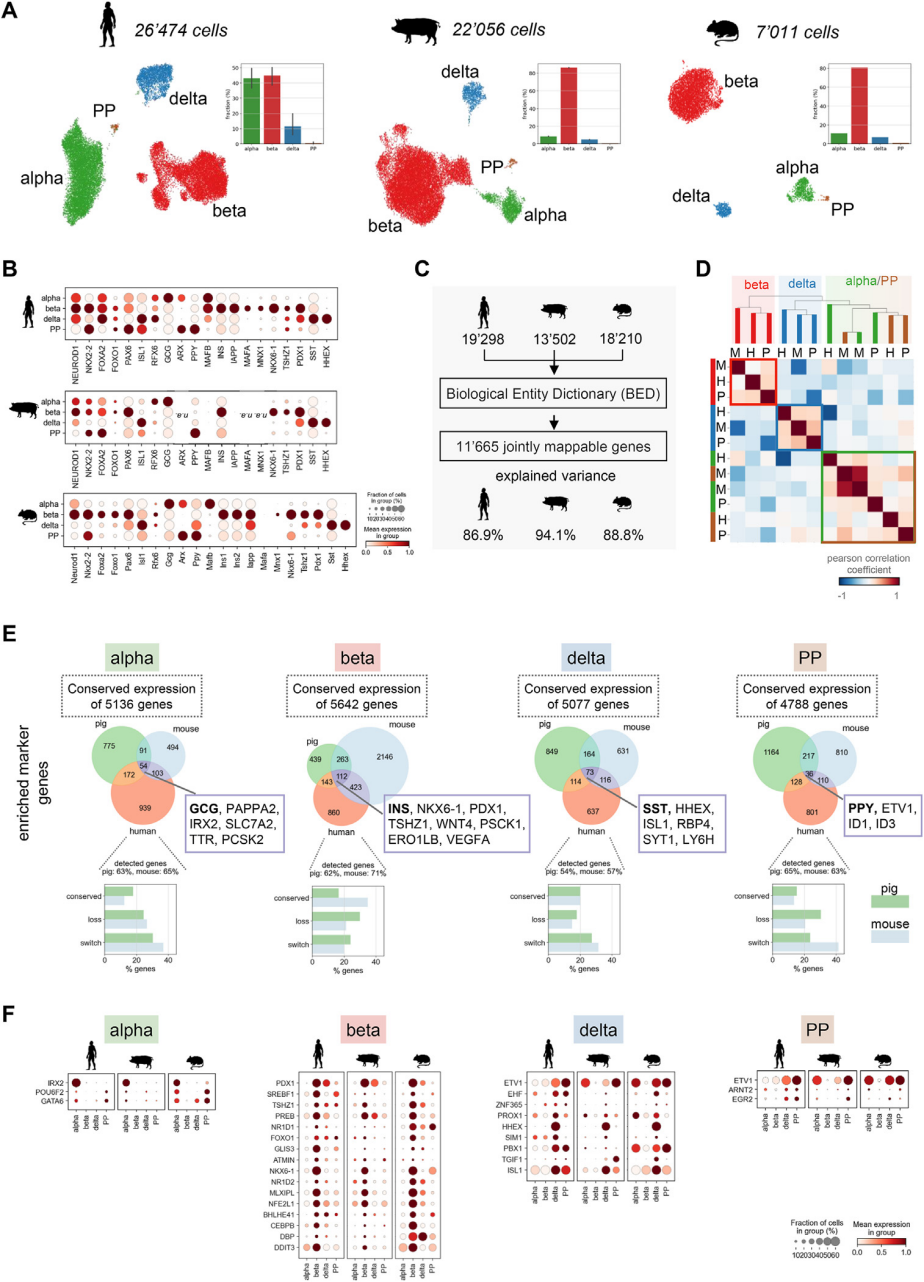
**Conservation of endocrine signatures in human, pig, and mouse islets.** A) UMAP plots of scRNA-seq data of human, pig and mouse pancreatic islets capturing all 4 major endocrine populations. Barplots show cell type compositions, which reflect islet composition *in vivo*. B) Expression of islet hormones and known endocrine and lineage transcription factors in human, pig and mouse endocrine cell types. Color intensity indicates mean expression in a cluster, dot size indicates the proportion of cells in a cluster expressing the gene. Expression is scaled per gene. *N. a.* means genes were not detected. C) Overview of gene orthologue mapping between species to assess conservation of the human transcriptional signature. Explained variance is the fraction of the total variance captured by the subset of mappable genes. D) Correlation matrix of gene expression indicates global conservation of transcriptional profiles of endocrine cell types across species. Cell types are grouped by hierarchical clustering. Pairwise correlation is computed in the principal component analysis space after excluding the top two variance components, which are entirely driven by cross-species variation (see also Figure S1C). α-, β- and δ-cells were subsampled to 2000 cells to balance cell type representation. E) Conservation of endocrine gene and marker expression. *Top:* Venn diagram showing overlap between species of enriched marker genes for each endocrine cell type. Only marker genes that are mappable across species are shown. Selected known overlapping cell type markers and number of genes with conserved expression are indicated. Enriched marker genes are defined as genes expressed in >5% of the cells of the corresponding cell type and showing increased expression versus all other cell types (log2-fold change>0.5). *Bottom:* Conservation of human enriched marker genes in pig and mouse cell types. % of human enriched marker genes expressed/detected is indicated. Conserved: enriched marker in same cell type as human; loss: detected but not an enriched marker; switch: enriched marker in different cell type than human. F) Expression of enriched and conserved transcription factors for each endocrine cell type in human, pig and mouse. Color intensity indicates mean expression in a cluster, dot size indicates the proportion of cells in a cluster expressing the gene. Expression is scaled per gene.

We sequenced >50′000 single cells from pancreatic islets isolated from 5 healthy human donors (age 22–74 years, male and female), a Göttingen minipig (2 replicates, age 3 years 8 months, female) and 3 mice (pooled, C57BLJ/6, age 23.5 weeks, male) to describe transcriptome-wide expression signatures of human endocrine cell populations and their conservation in animal models (Figure 1A, Figure S1A, B). To facilitate exploration and reuse of our data set we published it in the cellxgene portal (https://cellxgene.cziscience.com/collections/0a77d4c0-d5d0-40f0-aa1a-5e1429bcbd7e) and added it to the sfaira data zoo [27], which both follow the concept of FAIR data [26]. In all three species we identified the four main endocrine cell types: α-, β-, δ-, PP-cells. We captured a few rare *GHRL* positive ε-cells in the human, but not in pig and mouse samples, and therefore did not consider them for downstream analyses. Likewise, we excluded poly-hormonal cells as it is difficult to distinguish the profile of true polyhormonal cells from doublet cells (Supplementary Table 1). In human islets the ratio of α- and β-cells was relatively balanced, while in pig and mouse islets β-cells were most abundant (∼80%). These cell type frequencies are consistent with reported quantification in histological sections [28,29], which indicates our data is less confounded by technical artifacts than previous single-cell studies with low β-cell frequencies [14,23,25]. Human cells expressed established islet hormones and transcription factors defining endocrine cell identities. These expression patterns were conserved in pig and mouse clusters with a few known exceptions (Figure 1B). For example, the transcription factor *MAFB* was expressed in human α-, β- and δ-cells, but only in mouse α-cells. In pig, we detected low levels of *MAFB* in α-, β- and δ-cells similar to human islets as it was recently described in bulk expression profiles of sorted islet cells [20]. Such low detection levels are a general issue in RNA-seq studies of pig cells. The functional annotation of the pig genome is still less complete than for mouse and human genes, although continuity and quality of the reference sequence has been greatly improved [[30], [31], [32]]. Due to incomplete annotation of protein-coding genes, a subset of reads cannot be confidently mapped and are thus discarded. In our data this included the transcription factors *MAFA* or *ARX,* which were not detected in pig cells (Figure 1B). The lower mapping rate for pig sequencing data can limit the interpretability of genes that are not expressed.

To directly compare gene expression across species, we identified mappable gene orthologs using the Biological Entity Dictionary (BED) [33] tool (Figure 1C). Out of approximately 19'300 human, 13′500 pig and 18′200 mouse genes (annotated and detected), 11′665 genes were mappable across all three species. The 11′665 genes explained on average 90% of the total variance in each species (human = 87%, pig = 94%, mouse = 89%, Figure 1C). We computed pairwise correlation and clustering of cell type profiles in the principal component analysis (PCA) representation of the scaled and concatenated cross-species data to assess global transcriptional similarity of human, pig and mouse endocrine cell types (Figure 1D). We did not consider the two top-variance components, because they were almost entirely driven by cross-species variation (Figure S1C). Cell types correlated stronger among each other than among species, which indicates that globally cell type expression profiles were conserved (mean pearson's rho for α-cells = 0.15, for β-cells = 0.12, for δ-cells = 0.23, for PP-cells = 0.2, for human-cells = -0.15, for pig-cells = -0.26 and for mouse-cells = 0.02). Moreover, α- and PP-cells were closely related to each other and more distant to β- and δ-cells in all three species. During development mutual inhibition of lineage determinants promotes endocrine progenitors towards a α-/PP- or β-/δ-cell fate [34,35], thus, this developmental proximity of α-/PP and β-/δ-cells is reproduced in adult islets. Further, this suggests that developmental programs of endocrine subtype specification are conserved across species.

Next, we evaluated the overlap of gene expression between species in each cell type (Figure 1E, Supplementary Table 2). We found that on average 5′160 out of 11′665 mappable genes showed conserved expression in >5% of the cells in each endocrine cell type across species. This indicates that only 50–60% of genes expressed in human cell types are shared with their mouse and pig counterparts (Figure S1D). The majority of the other 40–50% were either only expressed in another cell type (“loss of expression”) or not expressed or detected. The remaining 5% were not expressed in human but detected in pig or mouse cells (“gain of expression”). For example, we detected high mRNA levels of *free fatty acid receptor 4* *FFAR4*, as well as calcium-sensing receptor *CASR* in human β-and δ-cells (Figure S1E). The expression of both genes was low or lost in mouse and pig β-cells but conserved in δ-cells (4.7% of cells in pig). In addition, mouse α-cells “gained” expression of *FFAR4* while pig α-cells “gained” *CASR.* Similarly, the synaptic protein *neuronal pentraxin-2* (*NPTX2)* was strongly expressed in human β-and δ-cells, all pig endocrine subtypes, but mostly lost or not detected in mouse cells. Instead, mouse cells expressed *neuronal pentraxin-1* (*NPTX1)*. The subtype expression pattern of the transcription factors *DNA-binding protein inhibitor ID1-4* was highly conserved in humans and pigs, but not in mice. These examples highlight cell type specific species differences in receptors and regulatory or signaling proteins relevant for islet function. As noted previously, not detected expression of a gene can be due to either biological species differences or technical factors such as genome annotation or sequencing depth. For validation, we compared our results to reported core genes derived from human and mouse bulk β-cell transcriptomes [36] (Figure S1F). From the 85.5% of core genes (8105/9474 core genes) captured within the 11′665 mappable genes, we found that 77% overlapped with those we identified as conserved between human and mouse β-cells. This indicates that our approach approximates conservation consistent with previous reports. Differences may be due to the distinct data types, how conservation is defined and or detection limits in scRNA-seq data.

Beyond global gene expression profiles, we focused on cell type enriched marker genes to approximate conservation of cell type-specific functions (Figure 1E, Supplementary Table 3). As a positive control, we verified that we identify established marker genes in all species, which included *GCG*, *IRX2* and *TTR* for α-, *INS*, *PDX1* and *NKX6-1* for β-, *SST* and *HHEX* for δ- and *PPY* for PP-cells. Surprisingly, of the remaining identified human marker genes only 5–10% were shared with both mouse and pig. The small overlap was not biased by one species, because the overlap with human markers was similar for mouse and pig markers. Overall, we observed that in all cell types less than 20% of the human markers were conserved, approximately 20% were expressed but did not appear as marker genes (‘loss’), and 30% marked other populations (‘switch’). The rest was not detected or expressed. We thus conclude that while critical identity and functional marker genes are conserved, cell type specific expression is evolutionarily more labile. We noted that, especially in mice, fewer enriched marker genes were detected and conserved in α- and PP-cells than in β- or δ-cells, which may be explained by the high similarity of mouse α- and PP-cell profiles.

Finally, we assessed the similarity of transcription factor (TF) expression patterns. TFs are key components of the gene regulatory networks that determine endocrine cell identity during development and maintain identity and function in adult islets. Thus, we considered TF patterns as another measure for proximity of animal models to humans (Figure 1F). We assumed, TF networks are most likely best evolutionary conserved within the shared marker genes and subset to shared TFs. Moreover, to quantify similarity we considered TF expression across cell types, because for modeling transcriptional regulation in a cell type, not only TF expression but also cell type-specificity should be conserved. Lastly, we computed a correlation measure that includes the mean expression as well as the fraction of cells expressing a TF in a cluster to leverage all information contained in single-cell data (Methods). With this approach, we observed that α- and β-cell TF patterns were better conserved between human and pig (pearson's rho = 0.97, p-value = 10^−7^ for α-cells, pearson's rho = 0.73, p = 10^−12^ for β-cells) than between human and mouse (pearson's rho = 0.73, p = 0.006 for α-cells, pearson's rho = 0.57, p = 10^−7^ for β-cells) (Figure S1G). α- And β-cell TF patterns also correlated stronger between human and pig than human and mouse when considering all TFs we identified as cell-type enriched markers in humans (Figure S1H), or, all TFs with conserved expression (not necessarily cell-type enriched) (Figure S1I). Conversely, for δ- and PP-cells, there were no pronounced differences between species when subset to conserved marker TFs (Figure S1G). Human and mouse δ- and PP- TF patterns correlated stronger within all enriched marker TFs (Figure S1H), while human and pig δ- and PP-TF patterns correlated stronger within all TFs with conserved expression (Figure S1I). Thus, this analysis suggests that α- and β-cell TF expression and likely target gene regulation is closer to human in pig than in mouse models.

### 2.2 β-Cell heterogeneity and phenotypic states in human islets

**Figure 2:**
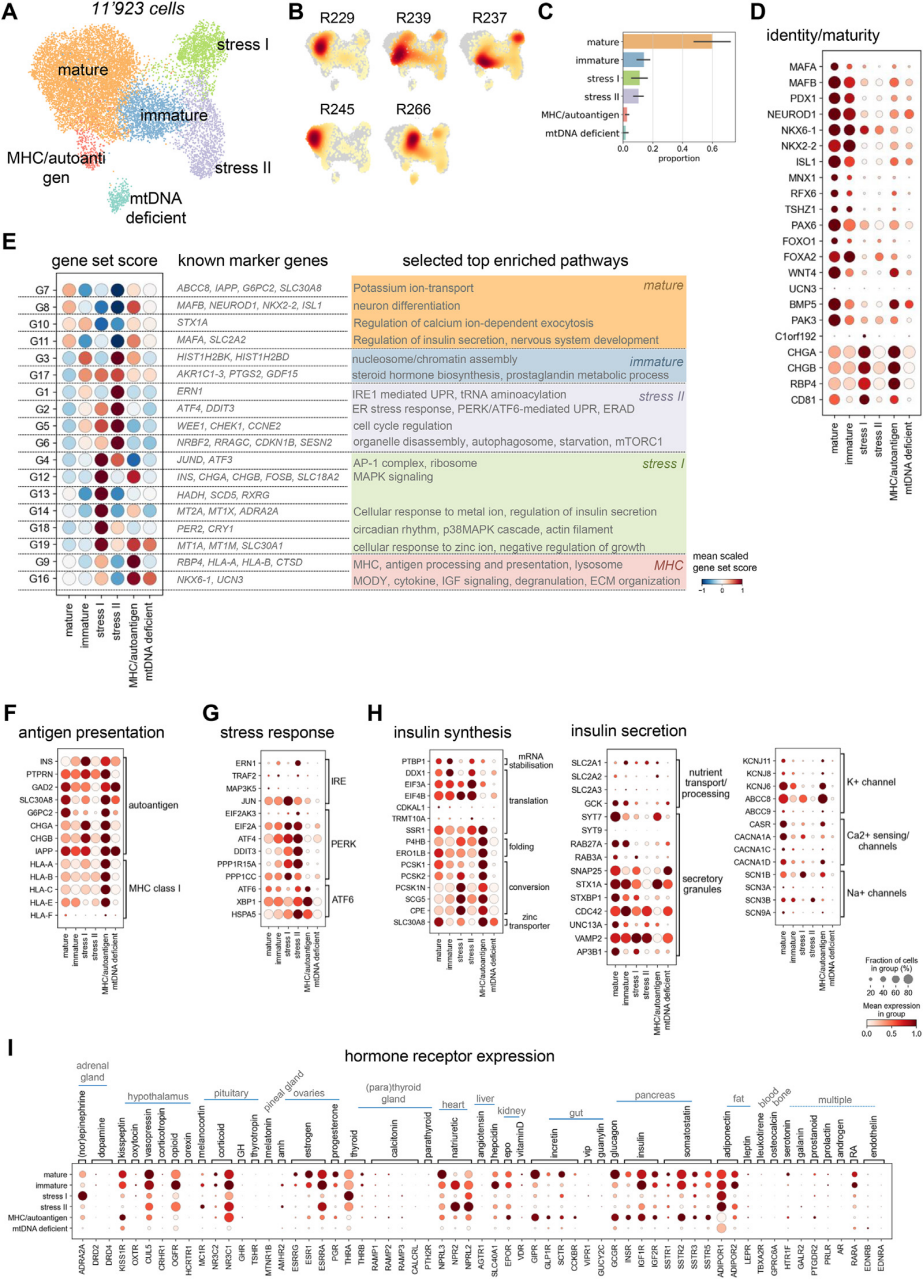
**Transcriptional β-cell heterogeneity and states in human islets.** A) UMAP plot of 11′923 human β-cells. Colors highlight clustering into six different β-cell states. B) Cell densities in UMAP space for five human donors shows that all β-cell clusters are represented by all donors. ID indicates donor ID for ADI IsletCore (see also Figure S1A). C) Fraction of cells per β-cell cluster. Error bar indicates donor variation. D) Expression of selected known β-cell identity and maturity markers. Color intensity indicates mean expression in a cluster, dot size indicates the proportion of cells in a cluster expressing the gene. Expression is scaled per gene. E) Gene sets capturing variation in human β-cells that describe biological processes. Gene sets are groups of highly correlated and/or anti-correlated genes identified using hierarchical clustering on the correlation matrix of the top 3000 variable genes. *Left:* Scaled mean score for each gene set per β-cell cluster. For each gene set selected known β-cell identity or functional marker genes are indicated. *Right:* Summary of selected enriched pathways for each gene set indicating biological processes associated to gene sets. Coloring indicates the highest scoring β-cell cluster. F–H) Expression of genes encoding MHC class I components and β-cell autoantigens (F), members of the three canonical ER stress response arms (G), and insulin synthesis and secretion pathways (H). Color intensity indicates mean expression in a cluster, dot size indicates the proportion of cells in a cluster expressing the gene. Expression is scaled per gene. I) Expression of receptors for the majority of circulating hormones in human β-cell clusters. The tissue or organ origin and the type of hormone are indicated. Only receptors detected in >5% of cells of any cluster are shown. Color intensity indicates mean expression in a cluster, dot size indicates the proportion of cells in a cluster expressing the gene. Expression is scaled per gene.

To understand β-cell heterogeneity in human islets, we clustered the human β-cells at higher resolution and identified six β-cell clusters (Figure 2A). These clusters did not form separated clusters, but rather connected states in the continuous β-cell manifold. All six clusters were represented in all five donors, but subtype composition varied across donors (Figure 2B,C). Approximately 60% of the cells formed a large cluster we annotated as *mature* β-cells, because they highly expressed canonical β-cell identity and maturity genes [37] (Figure 2C,D), and scored high for the β-cell hallmark pathway (Figure S2A). The other clusters made up less than 20% of all β-cells. Two clusters activated hallmark pathways associated with unfolded protein response, stress and apoptosis, which we therefore referred to as *stress I* and *stress II* cells (Figure S2A). Identity and maturity markers as well as β-cell hallmark scores decreased from the *mature* to the stress-clusters, which suggests a gradual loss of β-cell identity and maturity (Figure 2D). The state between the *mature* and the stress-clusters most resembled *immature* cells. In this intermediate state, pathways associated with the cell cycle and the PI3K-Akt-mTOR signaling axis were increased, which was previously reported to characterize less mature β-cells in mice [37,38] (Figure S2A). However, other reported markers of murine immature β-cells like *CHGB, RBP4* and *CD81* showed variable expression in the β-cell states that did not fully correlate with loss of maturity and identity markers (Figure 2D). We could not annotate the two remaining clusters based on this analysis, because the top scoring hallmark pathways were not related to an interpretable β-cell state, but described processes of other systems or tissues. Finally, we saw no strong upregulation of β-cell disallowed genes in any non-mature cluster compared to the *mature* cluster (Figure S2B). Thus, we identified clusters with established β-cell profiles, alongside novel transcriptional β-cell states.

β-cell-specific processes can be better captured when gene sets are identified with an unbiased, data-driven approach. We therefore clustered the 3000 most variable genes into groups of highly correlated and or anti-correlated genes (hereafter referred to as *gene sets*) and then related these gene sets to cellular processes based on known marker genes and pathway enrichment for interpretation (Figure 2E, Supplementary Table 4, Methods). This approach was previously described to identify *de novo* gene sets in single-cell data [39] and is commonly used in correlation network analysis [40]. In contrast to describing cell states with marker genes, it gathers genes into context-specific groups independent of the predefined cell states, i.e. the same set of genes can be activated in multiple cell states. We identified four gene sets (G7-8, 10–11) scoring high in *mature* β-cells that contain key markers and enriched pathways of β-cell identity, glucose sensing and insulin secretion (Figure 2E). These gene sets were decreased in the *immature, stress I and stress II* clusters.

Beyond canonical β-cell function, one gene set (G9) was enriched for factors involved in antigen processing and presentation including major histocompatibility complex (MHC) class I and lysosome. Cells scoring high for the MHC/antigen processing-associated gene set formed a small cluster we could not previously annotate and also highly expressed β-cell identity and function genes as well as reported β-cell autoantigens (Figure 2F). We therefore referred to the cluster as *MHC/autoantigen.* While healthy β- and other endocrine cells steadily present self peptides via MHC class I complex, hyperexpression of MHC class I genes has been observed in islets of T1D patients. Increased levels of MHC class I were suggested to contribute to aberrant antigen presentation and autoimmune-mediated β-cell destruction [41]. To confirm that this gene set captures biologically relevant β-cell features, we compared the *MHC/autoantigen* state to β-cells from T1D patients [42], and observed a high T1D β-cell-derived score in *MHC/autoantigen* cells (Figure S2D). *Vice versa*, T1D β-cells highly expressed MHC class I genes and activated the MHC/antigen processing gene set (G9) when compared to healthy β-cells (Figure 2E, F). Also in α- and δ-cells a small subset of cells scored high for this gene set, which suggests a similar MHC-high state exists in other endocrine cell types (Figure S2G). Besides an increased MHC and lysosomal gene expression, *MHC/autoantigen* scored low for a gene set enriched for ribosomal genes (G4) (Figure 2E). This may indicate reduced ribosomal biogenesis and translation. Consistently, the expression of multiple regulatory factors of translation (e.g. translation initiation factors) was decreased in the *MHC/autoantigen* state (Figure S2H). Moreover, *MHC/autoantigen* cells lowly expressed genes governing gene transcription including transcription initiation factors and subunits of RNA polymerase, which likely was linked to a reduced number of total genes expressed per cell (Figure S2H, I). G16, which contained β-cell markers *UCN3* and *NKX6-1*, also scored highest in the *MHC/autoantigen* cluster. However, low overall variance of the activation score for G16 indicated that the magnitude of the activation level differences was small and thus the gene set was similarly activated in all β-cells (Figure S2C). Together, this suggests the presence of rare β- as well as α- and δ-cells in healthy islets, which downregulate global transcription and translation, but maintain β-cell identity and enhance MHC class I-mediated antigen processing and presentation.

When insulin demand is high, the ER protein folding capacity of β-cells can be overwhelmed and misfolded proinsulin accumulates. To counteract the overload and its resulting stress, β-cells activate a UPR-mediated stress response [43,44]. For this adaptive UPR, also constitutive, low autophagy is considered important to remove the misfolded proteins and damaged organelles. We identified three gene sets, which captured these cellular stress response pathways and autophagosome and organelle disassembly (Figure 2E). All three gene sets were highly activated in the *stress II* cluster and a subset in the *stress I* cluster. Consistent with the gene set analysis, the three main global stress response arms - IRE, PERK and ATF6-mediated stress response-were differentially activated in the β-cell states (Figure 2G, Figure S2J). The PERK-arm was induced in the *stress I* and *stress II* cluster, ATF6 in the *stress II* and *MHC/autoantigen* cluster, while IRE was only active in the *stress II* cluster. *Stress I* cells scored high for further gene sets enriched for the stress-induced transcription factor *ATF3*, AP-1 complex, metallothionein, circadian rhythm (Figure 2E). Metallothionein and circadian rhythm genes are a part of the transcriptional program recently reported to be regulated by glucocorticoid signaling in human islets [45]. Glucocorticoid signaling has been associated with β-cell dysfunction and we therefore further compared the *stress I* profile to the transcriptional response glucocorticoid signaling induced. Like in glucocorticoid-treated islets, in *stress I* cells components of STAT and TGFβ-signaling as well as other islet growth factors including *Vascular endothelial growth factor A* (VEGFA) and *Platelet-derived growth factor subunit A* (*PDGFA*) were decreased (Figure S2K). Lastly, we annotated the remaining small cluster of cells as *mtDNA* *deficient* because mitochondria-encoded gene expression was low (Figure S2I). In this cluster most gene sets scored low, identity and maturity genes were decreased and also other data quality metrics were low (Figure 2D, Figure S2I). We therefore could not exclude that this cluster contained dying cells. In summary, our single-cell sequencing data captured distinct β-cell states that may reflect the transcriptional response to different stress factors. While maturity and identity markers and gene sets were not expressed in a large fraction of cells of non-mature β-cell states, stress-linked gene sets showed baseline activation in all β-cell states.

Finally, we sought to associate the distinct β-cell states with two key properties of β-cell function: insulin synthesis and secretion. We observed that all β-cell subpopulations expressed key genes of insulin synthesis (Figure 2H). Surprisingly, *stress I* and *MHC/autoantigen* cells expressed a higher level of *prohormone convertase 2* (*PCSK2*) than *prohormone convertase 1* (*PCSK1*) unlike the other β-cells. *PCSK*-genes encode enzymes that cleave pro-hormones including insulin and glucagon. Consistent with the increased *PCSK2* expression, also expression of *prohormone convertase subtilisin/kexin type 1 inhibitor* (*PCSK1N*) - a PCSK1 inhibitor - and the *Neuroendocrine protein 7B2* (*SCG5*) - a chaperone of *PCSK2*, which facilitates transport and function of *PCSK2* - was increased in the *stress I* and *MHC/autoantigen* clusters. In healthy human donors, *PCSK1* levels are reportedly higher in β-cells, while *PCSK2* levels are higher in α-cells [46]. A defective maturation of proinsulin is implicated in both T1D and T2D and plasma proinsulin to insulin ratio serves as a clinical index for β-cell dysfunction [[47], [48], [49], [50]]. Our analysis suggests that variable *PCSK* expression is part of the transcriptional programs turned on in β-cell substates, which eventually result in functional β-cell heterogeneity.

The activation of key insulin secretion processes was more heterogeneous (Figure 2H, Figure S2L). Relative to *mature* β-cells, multiple genes linked to glucose sensing, and secretory granules as well as ion channels were decreased in *immature, stress I and stress II* cells, but not in the *MHC/autoantigen* cluster. Beyond glucose and other nutrients, various circulating body hormones regulate insulin secretion. To identify the target β-cell states of these hormones we explored the expression of their cognate receptors (Figure 2I). Reduced receptor expression of known insulin secretion stimuli including other islet hormones, gut incretins, adipose tissue hormones or estrogen correlated with reduced expression of insulin secretion genes in *immature, stress I, stress II* clusters. In *stress I* cells decreased insulin secretion might be associated with increased *α-2-adrenergic receptor* (*ADRA2A*) expression and stimulation of inhibitory adrenergic signaling leading to reduced cAMP levels [51,52]. Consistently, the expression of several components of cAMP signaling was decreased in *stress I* cells (Figure S2M). In *stress II* and *immature* cells we observed a strong increase of *atrial natriuretic receptor 2* (*NPR2*) and the *Anti-Müllerian hormone receptor* (*AMHR2*). The effects of natriuretic peptides are still unclear, but insulinotropic and mitogenic effects on β-cells have been suggested [53,54]. To further corroborate that the described transcriptional heterogeneity is associated with functional heterogeneity we linked the β-cell states to electrophysiological measurements of exocytosis and ion channel activity in published single-cell “Patch-seq” data of human islet cells (“Patch-seq”: whole-cell patch-clamp measurements combined with RNA sequencing) [4]. To map the Patch-seq cells to our reference β-cell states, we represented them as activation scores of the β-cell gene sets (Methods). The 230 β-cells from healthy donors were similarly distributed across β-cell states and had similar marker expression and gene set activation profiles compared to our dataset (Figure S2N, O). As suggested from our transcriptional characterization, for *immature* and *stress II* β-cell decreased exocytotic function was measured compared to *mature* β-cells (Figure S2O). *Immature* cells also showed decreased Na + channel activity. No *MHC*-like and too few *stress I* cells were detected in the Patch-Seq data.

To confirm that the identified transcriptional β-cell states are robustly detected across study, age and sex we mapped β-cells of 9 single-cell studies (n = 54 donors) [9,13,14,23,25,[55], [56], [57], [58]] to our reference β-cell map in the representative gene set space (Figure S3A, Methods). Approximately 60% of cells mapped to the *mature* β-cell state, and 10–25% to *immature* β-cell state in all studies. Also *stress I, stress II* and *MHC/autoantigen*-like cells were consistently captured in multiple studies with a sufficiently large β-cell number (median >700 cells per donor). β-Cell state fractions were not significantly increased in female or male donors or correlated with age (Figure S3B, C), which indicates that the observed donor variation is not strongly linked to these variables in the integrated datasets.

Collectively, these results established that changes in β-cell function and maturation are reflected in the transcriptional profile of a cell and include activation of stress pathways and differential hormone receptor expression.

### 2.3 β-Cell maturation factors in human islets

**Figure 3:**
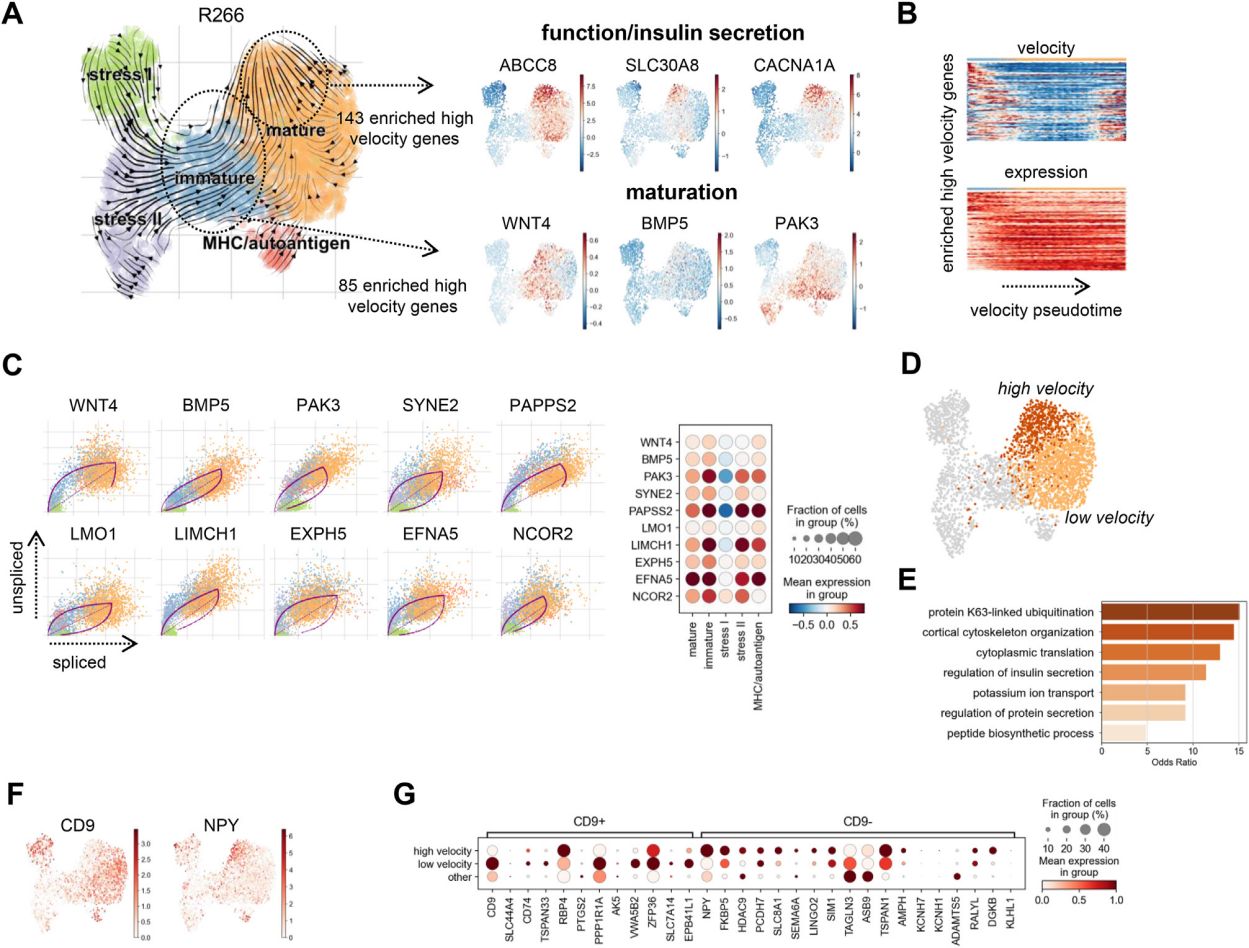
**Predicted transcriptional dynamics in human β-cell maturation and insulin secretion.** A) Cellular dynamics revealing areas of high induction and or repression of gene expression in β-cells of one human donor (R-ID 266). *Left:* Cell transitions are inferred from estimated RNA velocities and the direction of inferred movement plotted as streamlines on the UMAP. Colors indicate β-cell clusters. Circles highlight two areas of high velocity. Disconnected mtDNA deficient cluster was excluded. *Right:* UMAP showing the velocity of selected genes with increased velocity in the corresponding circled area. Top genes indicate induction of transcription of genes involved in β-cell function and insulin secretion. Bottom genes are associated with β-cell maturation. B) Velocity (top) and expression (bottom) of genes showing high velocity in immature β-cells along the cellular transition from immature to mature β-cells inferred from velocities. Cells were ordered by velocity pseudotime. Velocities and expression were scaled per gene. C) *Left:* Gene-resolved velocities of factors driving the transition from immature to the mature β-cell cluster. Purple lines indicate dynamics fitted with a full dynamical model. *Right: Dotplot showing mean velocity* per *β-cell cluster.* Selected known genes involved in β-cell maturation and potential novel genes important for maturation are shown. D) UMAP indicating two clusters of mature β-cells with high or low velocity. E) Selected top enriched Gene Ontology (GO) terms in high velocity genes of mature β-cells indicate induction of genes involved in insulin secretion. Gene enrichment was performed with EnrichR using a modification of the Fisher's exact test. F) Expression of two known markers of β-cell heterogeneity, CD9 and NPY, separates the two mature clusters in D). G) Expression of genes previously described to separate CD9+ and CD9- β-cells in high and low velocity mature β-cells. Color intensity indicates mean expression in a cluster, dot size indicates the proportion of cells in a cluster expressing the gene. Expression is scaled per gene.

**Figure 4:**
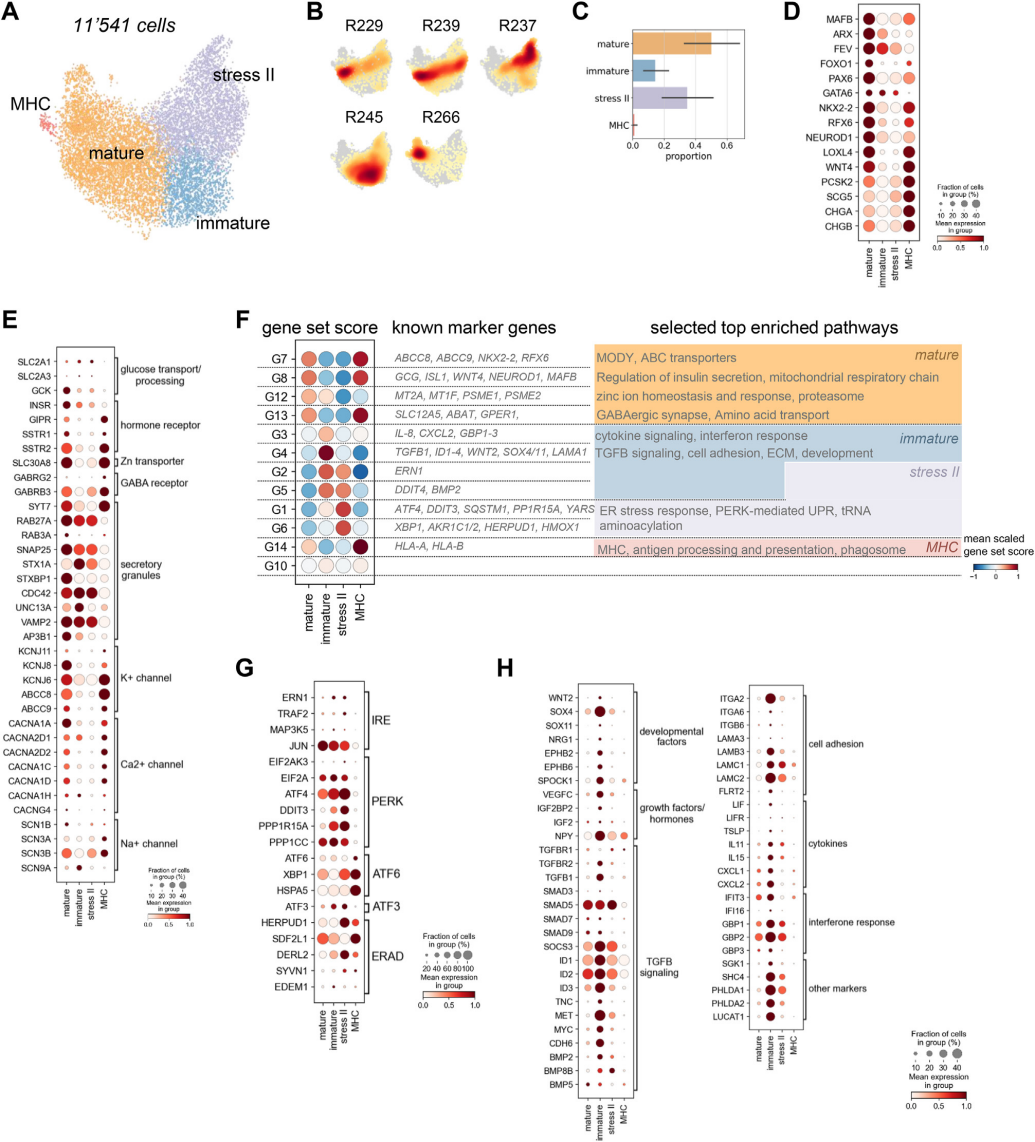
**Transcriptional α-cell heterogeneity and states in human islets.** A) UMAP plot of 11′541 human α-cells. Colors highlight clustering into four different α-cell states. B) Cell densities in UMAP space for five human donors shows that all α-cell clusters are represented by all donors. ID indicates donor ID for ADI IsletCore (see also Figure S1A). C) Fraction of cells per α-cell clusters. Error bar indicates donor variation. D-G) Characterization of α-cell clusters. D-E, G-H) Expression of selected known α-cell identity and maturity markers (D), functional markers (E), adaptive stress response genes (G) and genes involved in pathways describing immature α-cells (H). Color intensity indicates mean expression in a cluster, dot size indicates the proportion of cells in a cluster expressing the gene. Expression is scaled per gene. F) Gene sets capturing variation in human α-cells that describe biological processes. Gene sets are groups of highly correlated and or anti-correlated genes identified using hierarchical clustering on the correlation matrix of the top 3000 variable genes. *Left:* Scaled mean score for each gene set per α-cell cluster. For each gene set selected α-cell identity or functional marker genes are indicated. *Right:* Summary of selected enriched pathways for each gene set indicating biological processes associated to gene sets. Coloring indicates the highest scoring α-cell cluster.

For clinical research it is crucial to identify the transcriptional programs critical to induce or maintain a functional β-cell with high insulin biosynthesis and secretion capacity. Single-cell sequencing can reconstruct gene expression dynamics by RNA velocity inference [59,60] and thereby reveal factors underlying a transcriptional state change. We applied RNA velocity analysis to β-cells of each donor separately, since current velocity inference methods cannot account for batch- and or donor-variation. We then focused our analyses on one donor (ID R266), in which all β-states were well represented (Figure 3A), and confirmed the outcomes in the other four donors (Figure S4A). We identified two regions with high dynamics that captured *in silico* transcriptional state changes associated with β-cell maturation and insulin secretion, respectively. For the flow from *immature* to *mature* cells, we predicted high velocity for the signaling proteins *WNT4*, *BMP5* and *PAK3,* which are known markers of *mature* β-cells [38,61,62] (Figure 3A). This showed that maturity factors were actively transcribed in our immature cells, which suggests that the inferred dynamics may recapitulate aspects of β-cell maturation. Other genes with a similar dynamic behavior - i. d. high velocity in the *immature* cluster and high expression in the *mature* cluster - are additional putative maturation factors (Figure 3B,C, Supplementary Table 5). For example, we identified the co-regulatory *nuclear receptor co-repressor 2* **(***NCOR2*) as well as *LIM and calponin-homology domains 1* (*LIMCH1*) - a positive regulator of non-muscle myosin II promoting focal adhesion assembly - which, to our knowledge, have not been previously associated with β-cell maturation (Figure 3C). Further also *ephrinA5* (*EFNA5*), a well known factor of neurogenesis and potential regulator of insulin secretion in β-cells [63], showed high velocity in *immature* cells (Figure 3C). The inferred dynamic behavior of these genes was confirmed in the other donors (Figure 4A, B). We verified the transcriptional activity of the identified maturation-associated genes during β-cell maturation in single-cell data of human β-cell development from two studies [64,65] (Figure S5A-B, D-E). The expression of *WNT4*, *BMP5* and *PAK3* as well as *PAPSS2*, *LMO1*, *NCOR2*, *LIMCH1* and *EFNA5* and other identified factors was increased in immature β-cells compared to β-cell progenitors and precursors in fetal islets, which corroborates their role in β-cell maturation (Figure S5C, F).

Within the *mature* β-cell cluster, our analysis indicated a static and dynamic region of cells (Figure 3A,D). High velocity genes in the *mature* cluster were enriched for insulin secretion pathways and genes, which suggests that these dynamics describe transcriptional state changes from lower to higher insulin biosynthesis and or secretion activity (Figure 3D, E, Supplementary Table 5). The high and low velocity clusters were also separated by *CD9* and *NPY* expression (Figure 3F). CD9 has been proposed as a marker of functional β-cell heterogeneity, which together with ST8SIA1 separates β-cells into four subpopulations [66]. Additional markers of CD9^+^ and CD9^-^ cells were differentially expressed between high and low velocity *mature* cells (Figure 3G). Within this classification scheme, NPY is a marker for CD9^-^ ST8SIA1^+^ cells, which showed higher glucose-stimulated insulin secretion capacity consistent with the transcriptional activity in insulin biosynthesis and or secretion observed here. We found high and low velocity clusters with a similar marker expression profile also in the *mature* cluster of three out of the four other human donors (Figure S4C). In summary, our RNA velocity analysis predicts factors that promote possible state transitions in the continuous transcriptional β-cell landscape to and within mature β-cells. The predicted cellular flows from stressed/immature-like to mature and within mature cells indicate that these are likely interchangeable transcriptional states located along gene expression gradients and not stable β-cell subpopulations.

### 2.4 Human α-cell states

To describe molecular α-cell heterogeneity in human islets, we refined the α-cell clustering and identified four α-cell states, which were represented in all 5 donors (Figure 4A–C). As for β-cells we used known marker genes and pathways as well as data-driven gene sets to annotate and characterize the α-cell states (Figure 4D–F). We annotated a cluster of approximately 50% of the α-cells as *mature* (Figure 4A–F). The *mature* cells highly expressed known α-cell or endocrine identity and maturation factors as well as glucose transporters, hormone receptors, secretory-granule linked genes and ion channels important for α-cell function (Figure 4D,E). These key markers as well as pathways linked to α-cell function including glucagon secretion, insulin regulation and the mitochondrial respiratory chain were also captured by four α-cell gene sets (G7-8, 12–13), which were activated in the *mature* α-cells (Figure 4F, Supplementary Table 4). More than 30% of α-cells showed an increase of PERK-mediated stress response genes and gene set scores and a decrease of α-cell identity and function factors similar to *stress II* β-cells and were therefore annotated as *stress II* α-cells (Figure 4F,G). 1% of cells were MHC-like α-cells with increased MHC gene expression and gene set activation (Figure S2G). The remaining α-cell cluster had an *immature* or precursor-like profile (Figure 4F,H). Multiple developmental markers including *SOX4, SOX11, NRG1, ID1-4*, *EPHB2* and *EPHB6* were increased, while α-cell function genes were decreased. *Immature* α-cells also activated gene sets enriched for TGFβ signaling, cell adhesion, ECM components, cytokines and interferon response (G2-5) as well as several direct transcriptional targets of the TGFβ signaling or interferon response pathway (Figure 4F,H). We verified activation of these gene sets in endocrine precursors and *immature* α-cells in single-cell data of human pancreatic development [64,65] (Figure S5A, S6A,B). Fetal FEV^+^ endocrine and α-cell precursors scored higher than α-cells for the *immature* and TGFβ-linked gene sets, but not for the inflammatory responses (Figure S6A, B). In addition, a subset of the identified markers of *immature* α-cells were expressed in fetal precursors and α-cells, which together confirms that parts of the profile of the *immature* adult α-cell state resembles that of developing α-cells (Figure S6B). Stress-linked α-cells formed less distinct clusters than stress-linked β-cells (Figure S6C), which indicates that α-cells were transcriptionally more homogenous and elicited a smaller stress response.

Finally, we leveraged published Patch-seq data to link the observed transcriptional states to α-cell electrophysiology [4] (Figure S6D-G). Cells from healthy donors mapped to the *mature, immature* and *stress II* reference α-cell states, hence these transcriptional states are robustly detected in different human data sets and donors (Figure S6D, E). Like in our reference map, *immature* cells had increased expression of developmental markers, TGFβ signaling and interferon response genes (Figure S6F). *Stress II* cells upregulated a canonical stress response (Figure S6F). In both *immature* and *stress II* cells Na^+^ and Early Ca^2+^ currents were decreased, while the other electrophysiological parameters were unchanged (Figure S6G). Molecular heterogeneity described by a set of marker genes was already associated with differences in Na^+^ and Early Ca^2+^ currents by [4]. Here, we established that two transcriptionally distinct states may underlie this functional α-cell heterogeneity highlighting two potential routes that lead to decreased function.

### 2.5 Cross-species mapping of human α- and β-cell heterogeneity

**Figure 5:**
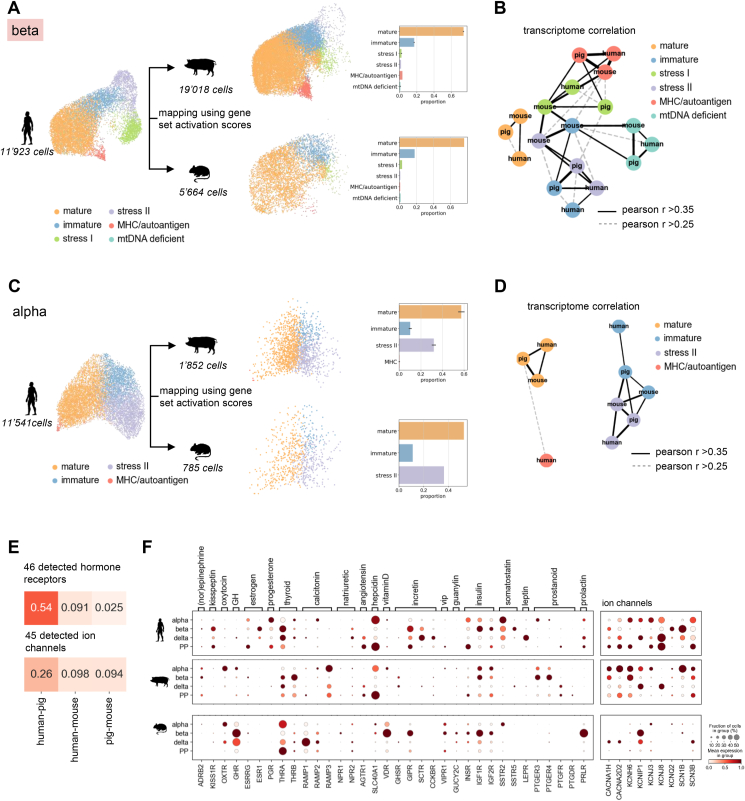
**Cross-species mapping of α- and β-cell states.** A-D) Cross-species mapping of α- and β-cell states. A,C) Representation and cross-species mapping of β- (A) and α-cells (C) by gene set activation scores. UMAP plot (left) shows human cells, where each cell is represented by an activation score of the corresponding cell gene sets. Pig and mouse cells were mapped to the human reference data through projecting on the human gene set representation. Embedding and labels are mapped using the Scanpy ingest functionality (see Methods). The barplot indicates the frequencies of mapped clusters for pig and mouse. B, D) Graph showing global transcriptome correlation of β-(B) and α-(D) cell clusters across species. Edge weights indicate pearson correlation coefficient (see also Figure S4B). Nodes are colored by β-cell clusters. E) Pairwise correlation of the expression pattern across endocrine cell states computed using detected hormone or hormone-like receptors (top) or ion channels (bottom). α- And β-cells were subset to mature state. List of hormone receptors was manually curated. List of ion channels contains calcium, sodium, potassium and transient receptor potential ion channels. Pearson correlation is computed using the harmonic average of mean expression and fraction of cells expressing a gene in a group across all cell types (see Methods). Pearson correlation coefficient is indicated. F) Expression of selected hormone receptors (left) and ion channels (right) showing differential expression patterns in endocrine cell states across species. α- And β-cells were subset to mature state. Hormone and peptide ligands for receptors are indicated. Color intensity indicates mean expression in a cluster, dot size indicates the proportion of cells in a cluster expressing the gene. Expression is scaled per gene.

Gene sets are a data representation, which captures the human α- and β-cell biology but removes species- or batch-specific details and overcomes technical artifacts like the limited annotation and capture rate in pig. If one assumes that the subset of mappable genes is sufficient to indicate activation of the full gene set, the gene set space corresponds to normalizing the data per functional gene set unit. To assess conservation of the human α- and β-cell states, we represented each cell as an activation score of the human α- or β-cell gene sets, respectively, and projected mouse and pig cells to the human reference map (Figure 5A,C).

The majority of pig and mouse β-cells mapped to the *mature* human reference cluster and scored high for the identified maturity gene sets (Figure 5A, Figure S7A). The mapped *mature* cells highly expressed β-cell identity and maturity markers and their gene expression profiles strongly correlated with the human *mature* profile (Figure 5B, Figure S7B, C), which validates our gene set-based mapping strategy. A smaller cluster of pig and mouse cells resembled *immature* cells and showed decreased levels of maturity gene set scores and markers (Figure 5A, Figure S7A, B). Moreover, a small fraction of cells mapped to the *stress I* and *stress II* references (Figure 5A). In mice, the expression profiles of *immature, stress I* and *stress II* correlated stronger with each other, cells clustered more tightly, and activation level differences of markers and gene sets were smaller than for human and pig β-cells (Figure 5B, Figure S7A-D). For example, multiple stress response genes including *ATF3*, *DDIT3*, *PPP1R15A*, *HSPA1B*, *DNAJB1, SYNV1, DERL3, FKBP11, SXRN1* were expressed in most *mature* and non-mature mouse β-cells, while they were more specifically increased in *stress I* or *stress II* clusters of pig and human β-cells (Figure S7D). Hence, mouse β-cells were more homogeneous than human and pig β-cells and adopted a *mature* or immature-like state with high basal expression of stress-response factors but not a distinct stress-associated state. Lastly, we identified in both pig and mouse β-clusters that mapped to the *MHC/autoantigen* human β-cells, which activated the MHC/autoantigen-associated gene set (G9) and decreased the ribosome/translation-associated gene set (G4), and whose profiles strongly correlated with their human counterparts. This indicates that the *MHC/autoantigen* β-cell state is evolutionarily conserved.

Pig and mouse α-cells mapped to *mature, immature* and *stress II* reference states and were similarly distributed as human α-cells (Figure 5C). In *mature* cells identity and maturation markers as well as maturity gene set activation were conserved and their transcriptomes correlated across species (Figure 5D, Figure S7E-G). The transcriptomes of *immature* and *stress II* cells correlated strongly across and within species (Figure 5D, Figure S7F). Like in human α-cells, *immature* cells had increased activation of TGFβ-associated genes and a subset of other developmental factors (Figure S7H). However, we did not detect increased cell adhesion/ECM factors or an inflammatory response in pig and mouse cells. Similar to β-cells, in mouse expression level differences of stress-associated genes were smaller and *stress II* cells less distinct from mature/immature cells than in human and pig (Figure S7G, I). To confirm that the cross-species comparison and observed states are robust across datasets we mapped α- and β-cells of three additional healthy mice [67] to our human references (Figure S8, Methods). For both α- and β-cells, detected states and state fractions (Figure S8A,C) and gene set activation (Figure S8B,D) were consistent with results observed for the mouse data used in this study. Together, our analyses suggest that the spectrum of human transcriptional α- and β-cell heterogeneity including stress-associated states were better captured in our pig than mouse data.

Finally, we investigated conservation of the transcriptional profile of human *mature* states. We first focused on mappable genes within the α- and β-maturity gene sets, respectively. Of these genes more than 60% were conserved in *mature* β-cells and more than 70% were conserved in *mature* α-cells of pigs and mice (Figure S7J). Moreover, putative human β-cell maturation factors identified by RNA velocity analysis were expressed in mouse and pig *mature* β-cells (Figure S7K). Finally, to approximate conservation of hormone/peptide signaling and excitability in mature cells we explored hormone or hormone-like receptors and ion channels in mature α- and β-cells and the other endocrine cell types δ- and PP-cells. Overall, the expression patterns across endocrine cell types of both detected hormone receptors and ion channels (calcium, potassium, sodium and transient receptor potential ion channels) correlated stronger between human and pig than human and mouse (Figure 5E). Differentially expressed receptors in mouse when compared to human islets included for example *the prolactin receptor (PRLR), leptin receptor (LEPR), Vitamin D receptor (VDR), growth hormone receptor (GHR), Natriuretic peptide receptor A (NPR1), Estrogen receptor 1 (ESR1), Progesterone receptor (PGR), Vasoactive intestinal polypeptide receptor (VIPR), guanylate cyclase-C receptor (GUCY2C), secretin receptor (SCTR), prostanoid receptors (PTGER3, PTGER4, PTGFR)* as well as *ferroportin (SLC40A1)* (Figure 5F). *PRLR, VDR, VIPR, NPR1, GUCY2C* and *GHR* were highly expressed in mouse but low or absent in pig and human mature β-cells and instead detected in other endocrine cell types. Similarly, *ADRB2*, *PGR* and *ESR1* were expressed in human but not in mouse β-cells, and, *ADRB2* but not *PGR* and *ESR1* was also detected in pig β-cells. We confirmed that all of these receptors were unique or enriched in mouse or human β-cells, respectively, in bulk β-cell transcriptomes of human and mouse islets [36]. Surprisingly, pig β- and α-cells expressed *PTGER3* and *PTGER4*, which in mice have been reported as β-cell dedifferentiation markers. Especially, *PTGER3* was strongly upregulated in STZ-treated diabetic β-cells (Figure S7L). In humans, *PTGER3* and *PTGER4* were expressed in α-cells. Ion channels with differential expression in mouse and human β-cells included potassium channel *KCNJ8*, sodium channel *SCN3B* and calcium channels *CACNA1H* and *CACNA2D2* (Figure 5F). *KCNJ8* was expressed in all human endocrine cell types and in pig δ-cells but not detected in mice. *SCN3B, CACNA1H* and *CACNA2D2* were expressed in all human and pig cell types, but only in mouse δ-cells. Like prostanoid receptors, these channels were increased in diabetic β-cells of STZ-treated mice (Figure S7L).

In summary, the identified species-specific expression patterns of hormone receptors and ion channels suggest that these functional genes are better conserved in pig than mouse endocrine cells. Moreover, they exemplify the value of this data resource to explore differences between human and two commonly used animal models.

## 3 Discussion

Our single-cell data of human, pig and mouse endocrine islet cells is a foundational resource for advancing our understanding of human endocrine heterogeneity and its conservation in clinically relevant animal models. We characterized a compendium of human transcriptional α- and β-cell states, which represent a reference to investigate endocrine cell function, maturation and disease-associated phenotypes. The distinct non-mature α- and β-cell states (immature/stress/MHC) do not necessarily represent cells found as such *in vivo* in healthy patients, but likely have been induced during tissue isolation, processing, storage and transport. Moreover, the *in silico* predicted transcriptional dynamics indicate that these states are likely physiological and interchangeable states different from stable subpopulations, which transition only upon specific signaling cues and can be followed by lineage tracing [38]. Nevertheless, the captured cell states model mature, functional α- and β-cells as well as different types of endocrine cell stress. For example, our analyses revealed novel putative β-cell maturation markers (e.g. *NCOR2, LIMCH1, EFNA5*) and a distinct, conserved immature α-cell state with increased expression of developmental markers (e.g. *WNT2, SOX4, SOX11*), members of the TGF-β signaling pathway (e.g. *TGFB1, ID1-3, SOCS3, TNC*), integrins (e.g. *ITGA2, ITGA6*) and a cytokine response. Endocrine precursor cells of fetal human islets share parts of the transcriptional profile of immature-like α-cells [64]. Stressed α- and β-cells differentially express markers of hormone biosynthesis and secretion and regulatory hormone receptors and match cells with divergent electrophysiological properties, which may mirror aspects of the pathological phenotype reported for type 1 and type 2 diabetic islet cells [68]. We found that β-cells responded diversely to the multiple exogenous stressors they were exposed to during processing and described three distinct states linked to stress. These included a rare, but conserved β-cell state with a reduced expression of factors governing general transcription and translation, but increased MHC-class I and antigen expression. This suggests that in a state of high stress, in which global transcription is diminished, β-cells can maintain expression of identity genes and enhance antigen presentation activity, of which the latter is a gene program also observed in β-cells of T1D patients. Overall, we hope that this comprehensive human islet cell map will guide future hypotheses on the control and molecular basis of functioning islet cells and their response to stress, while also informing the path to successful therapeutic reestablishment of islet cell function in diabetic patients.

Despite correlation of whole transcriptional profiles and TF expression patterns of cell states, the conservation of human gene expression is surprisingly low (50–60%). We may have underestimated conservation due to detection limits inherent to single-cell RNAseq data and, for pig, due to the sparser coverage and annotation of the genome. Nonetheless, our findings suggest that large parts of gene expression patterns are evolutionarily labile, while important identity and functional marker genes and TF expression patterns are conserved. This is consistent with previous reports that showed similarly low conservation of cell type enriched genes between human, mouse and zebrafish [69]. These species-differences likely do not result in altered functional or phenotypic cell states, but they can become relevant in animal studies designed to identify pathological programs and clinical targets.

Our analyses provide evidence that pigs can be a surrogate model of gene expression relevant for human endocrine cell function. We showed that, overall, expression and cell type-specificity of regulatory units like TFs, hormone/peptide signaling and cell excitability are better mirrored in pig than mouse islet cells. For example, mature human and pig α- or β-cells shared functional regulators not observed in mouse, which included the TFs *ID1-4,* the surface hormone receptors *ADRB2* and *PTGER3* and the ion channels *SCN3B*, *CACNA1H* and *CACNA2D2*. These examples correspond well with reported differences between human and mouse β-cells [36], and illustrate the value of this data resource to reveal species-specific expression of targets governing glucose sensing and hormone secretion and to complement existing data sources of humans and mice. Finally, we observed that in our data the extent of human transcriptional α- and β-cell heterogeneity - especially expression gradients of stress-associated genes - is better conserved in pigs than in mice. While α- and β-cells of all three species adopted mature and more immature-like states, only human and pig cells formed distinct stressed states. In mice, stress-response factors (e.g. *DDIT3, PPP1R15A, DERL3, ATF3, DNAJB3, HSPA1B*) were expressed more homogeneously with a high basal level even in the mature state.

Altogether, our cross-species islet map provides a framework for investigating the transcriptional programs of human endocrine cells and represents a FAIR data resource [26] that can inform future studies where mouse and pig will fail to model human islet biology.

## 4 Methods

### 4.1 Cell sources

Primary human islets were obtained from the IsletCore facility (Edmonton, AB, Canada) with informed consent**.** Detailed donor information can be accessed via https://www.epicore.ualberta.ca/isletcore/ using the R-IDs indicated for each donor in Figure S1.

A female retired breeder Göttingen minipig (age: 3 years, 8 months) was purchased from Ellegaard (Denmark) and housed under standard conditions (19–23 °C; 40–70% relative humidity; 12:12 h day/night cycle). Pancreas retrieval and islet isolation was conducted as previously described [70]. Briefly, pancreas was preserved in Custodiol®- HTK solution for 2.5 h (cold Ischemia time). For islet isolation cold perfusion solution (Corning®, NY, USA) with Collagenase NB8 (4 U/g tissue), neutral protease (0.4 U/g tissue; both Serva, Heidelberg, Germany) and 100 mg DNase (Roche Diagnostics, Mannheim, Germany) were infused into the pancreatic duct. The digestion was performed by a modified Ricordi method at low temperature (34 °C) and with minimal mechanical force. Islets were separated from exocrine tissue by centrifugation on a discontinuous Ficoll (Sigma–Aldrich, Taufkirchen, Germany) density gradient in a COBE 2991 cell processor (Terumo BCT). After purification, islets were cultured in CMRL 1066 medium supplemented with 10% heat inactivated FBS, 100U/mL penicillin, 0.1 mg/ml streptomycin (all Gibco®, Darmstadt, Germany) and 32.5 mM l-glutathione (Sigma–Aldrich, Taufkirchen, Germany) at 37 °C in a 5% CO_2_ incubator.

### 4.2 Single-cell suspension

To obtain a single-cell suspension of human and pig islets, 60 islets were hand-picked into a 1.5 ml Eppendorf tube, pelleted (280 g, 1 min), washed with PBS (minus Mg or Ca, Gibco) and digested with Tryp-LE (Gibco) at 37 °C for 12 min. During the incubation step with Tryp-LE, islets were mechanically disaggregated with a 1 ml pipet tip every 2–3 min. The digestive reaction was then stopped by adding FACS-buffer (PBS, 2% FCS, 2 mM EDTA) and cells were pelleted (280 g, 3 min). Cells were stained with trypan blue to visualize dead cells and counted with a hemocytometer.

### 4.3 Single-cell sequencing

Single-cell libraries were generated using the Chromium Single Cell 3′ library and gel bead kit v2 (PN #120237) from 10x Genomics. Briefly, we targeted 10′000 cells per sample by loading 16,000 cells per sample onto a channel of the 10x chip to produce Gel Bead-in-Emulsions (GEMs). This underwent reverse transcription to barcode RNA before cleanup and cDNA amplification followed by enzymatic fragmentation and 5′adaptor and sample index attachment. Libraries were sequenced on the HiSeq4000 (Illumina) with 150 bp paired-end sequencing of read2.

### 4.4 Preprocessing and quality control of scRNA-seq data

For human and pig single-cell samples, the CellRanger analysis pipeline (v2.0.0) provided by 10x Genomics was used to demultiplex binary base call (BCL) files, to align and filter reads and to count barcodes and unique molecular identifiers (UMI). Barcodes with high quality were selected based on the distribution of total UMI counts per cell using the standard CellRanger algorithm for cell detection. All downstream analyses were run with python3 (v>=3.5) using the Scanpy package [71] (v>=1.4, https://github.com/theislab/scanpy) except stated differently. Python package versions that may affect numerical results are indicated in the available jupyter notebooks (See Data and Code availability). Genes with expression in less than 20 cells were excluded. Low quality or outlier cells were removed if the fraction of mitochondria-encoded counts was above 20%; (2) and based on total UMI counts and total genes. In human samples, thresholds were defined per sample after visual inspection of the total UMI count and total gene distributions as recommended [72] (for threshold values, see Data and Code availability and provided analysis notebooks). Cell-by-gene count matrices of all samples of one species were then concatenated to a single matrix. To account for differences in sequencing depth, UMI counts of each cell were normalized using the SCRAN algorithm [73] as implemented in the scran R package [74] and values were log-transformed (log (count+1)). Sample differences in human and pig samples were corrected as recommended [75] using the python implementation of ComBat [76] (https://github.com/brentp/combat.py) adopted by Scanpy (pp.combat) with default parameters and specifying each sample as one batch. Zero values were kept as zero even after correction to avoid spurious sample-to-sample differences around zero.

For mouse single-cell data [11] the filtered and annotated raw count matrix was downloaded from the Gene Expression Omnibus (GEO) (GEO accession number: GSE128565). The raw count matrix was filtered by subsetting to cells present in the filtered count matrix. Counts of each cell were normalized by total counts of that cell (pp.normalize_total with *exclude_highly_expressed* = True). Highly expressed genes (genes with more than 5% of total counts in a cell) were excluded from total counts for each cell before normalization. Counts were then log-transformed (log (count+1)).

These count matrices were used as input for further analyses unless indicated. Data from each species was analyzed separately until cross species mapping described below. Custom scripts with source code for all analyses of scRNA-seq data are available as jupyter notebooks in a github repository (https://github.com/theislab/2022_Tritschler_pancreas_cross_species) and the scRNA-seq data can be explored in the cellxgene data portal (https://cellxgene.cziscience.com/collections/0a77d4c0-d5d0-40f0-aa1a-5e1429bcbd7e).

### 4.5 Single cell manifolds, clustering and annotation

The manifolds and clusterings for the human, pig and murine endocrine cells and the human α- and β-cells were computed separately by performing the following steps. A single-cell neighborhood graph (kNN-graph was computed on the top principal components: 50 first for endocrine cells and α-cells, 25 first for β-cells) using 15 neighbors. Genes with expression in less than 10 cells were excluded. To calculate the principal components top highly variable genes were used as identified by the highly_variable identification function in Scanpy (pp.highly_variable, top 4000 for mouse endocrine cells, top 2000 for others). Clustering was performed using louvain-based clustering [77] as implemented in louvain-igraph (v0.6.1 https://github.com/vtraag/louvain-igraph) and adopted by Scanpy (tl.louvain). The resolution parameter was varied in different parts of the data manifold to account for strong changes in resolution (for details, see Data availability and provided analysis notebooks). For single-cell manifolds and visualization UMAP was run as recommended [78] and adopted by Scanpy. From the initial data mono-hormonal endocrine cells were annotated based on expression of genes encoding the four main islet hormones: insulin for β-cells, glucagon for α-cells, somatostatin for δ-cells, pancreatic poly-peptide for PP cells and ghrelin for epsilon cells. Clusters expressing known markers of non-endocrine cells (for example *SPP1* for ductal cells*, PRSS2* for acinar cells, *PLVAP* for endothelial cells, *PTPRC* for immune cells or *COL1A1* for fibroblasts and stellate cells), cells identified as doublets based on scores computed with the Scrublet algorithm [79] (v0.2.1, https://github.com/AllonKleinLab/scrublet) and co-expression of marker genes, and polyhormonal cells expressing multiple pancreatic hormones were excluded. α- And β-cell states were annotated as described in the main text. Clusters expressing the same hormones, markers or gene sets (α- and β-cell states) were merged (see also Data availability and provided analysis notebooks).

### 4.6 Gene orthologue mapping

To identify the genes mappable between species we used the R-based biological entity dictionary (BED). Briefly, first, ensembl gene names of pig samples were converted to human and mouse ensembl gene names, and then subset to the genes shared across species, detected in the data and with an ID set as preferred by the BED tool. For genes that did not map 1:1 between pig and human or pig and mouse (approximately 5% of all genes) the gene with the maximal expression in the corresponding species-data was kept. The list of mappable and detected genes is provided in the github repository (https://github.com/theislab/2022_Tritschler_pancreas_cross_species/BED_mapping_genes.csv).

### 4.7 Marker gene detection and comparison

Enriched marker genes of endocrine cell types were identified by comparing the mean expression of cells of one cell type to the mean expression of cells in all other cell types within each species. Genes that were expressed in at least 5% of the cells of the cell type and were increased by at least 1.4 fold (log_2_ (fold change) > 0.5) were defined as enriched marker genes.

### 4.8 Correlation based-gene sets of human α- and β-cells

Gene sets of human α- and β-cells were identified by clustering the top 3000 variable genes based on their pairwise–pearson correlation values across human α- or β-cells, respectively, as previously described in [39] to identify *de novo* gene sets. Genes detected in less than 20 α-/β-cells were excluded. Clustering was performed using Ward's method and euclidean distance as implemented in the scipy python package [80] (v.1.5.4). Functional enrichment of gene sets was performed as described below. Gene sets with very low average correlation (<0.005) were excluded from downstream analyses.

### 4.9 Similarity of gene expression patterns

Similarity of gene expression patterns was estimated by pearson correlation coefficients of gene expression across cell types or states to account for cell type or state-specificity. To leverage all information gained from single cell resolution, Pearson correlation coefficients were computed using the harmonic average of mean expression and fraction of cells expressing a gene in a group across all cell types. To account for differences in detection limits and sequencing depth the fraction of cells expressing a gene in a group was normalized to the mean fraction per group and species.

### 4.10 Pathway and transcription factor sources and pathway enrichment

Pathway enrichment of gene lists and sets was performed using EnrichR [81] as adopted by the enrichr functionality in the gseapy package (https://github.com/zqfang/GSEApy/). To evaluate hallmarks and stress pathway activations, hallmark and ontology gene sets were downloaded from the Molecular Signatures Database v7.2 of the Broad Institute. To identify transcription factors within gene lists a list of human transcription factors was downloaded from the Human Transcription Factor Database [82] (http://bioinfo.life.hust.edu.cn/HumanTFDB, v1.01).

### 4.11 Gene set activation and cell scores

Gene set or pathway activation in a cell was computed using the cell scoring function described by [83] and implemented in Scanpy (tl.score_genes). Briefly, the activation score of a cell is the average expression of genes of the gene set in a cell subtracted with the average expression of genes of a randomly sampled background set with expression values within the same range.

### 4.12 Characterization of T1D β-cells

Raw count matrices of cells from healthy and T1D patients generated by [42] were downloaded from GEO (Accession number GSE121863). Genes expressed in less than 10 cells were excluded. Raw counts of each cell were normalized by total counts of that cell not considering highly expressed genes for the total count normalization factor of a cell (pp.normalize_total with *exclude_highly_expressed* = True) and log-transformed (log (count+1)). Mono-hormonal β-cells were identified by iterative clustering and annotation as described above. The T1D β-cell score was computed based on the top 50 differentially expressed genes between β-cells from healthy and T1D donors (Welch's t-test, tl. rank_genes_groups).

### 4.13 Characterization of fetal human precursor α- and β-cells

Raw count matrices generated by [64] were downloaded from the data visualization center descartes (https://descartes.brotmanbaty.org/bbi/human-gene-expression-during-development/). The rsd-file was loaded into R and an AnnData object was generated for downstream analysis with the rpy2 (v3.3.5, https://github.com/rpy2/rpy2) and anndata2ri (v1.0.4, https://github.com/theislab/anndata2ri) python packages. Raw count matrices generated by [65] using the 10X Genomics technology were downloaded from OMix (https://bigd.big.ac.cn/omix/) using the identifier OMIX236. An AnnData object was generated for downstream analysis.

Both datasets were processed and analyzed following the same steps: Genes expressed in less than 10 cells were excluded. Raw counts of each cell were normalized by total counts of that cell. Highly expressed genes in a cell were not considered for the total count normalization factor of that cell (pp.normalize_total with exclude_highly_expressed = True). Counts were then log-transformed (log (count+1)). Pancreatic cell types and endocrine clusters were identified by clustering and annotation using markers described above. To distinguish epithelial from mesenchymal cell clusters the markers EPCAM and VIM were used. In [65], to detect neuronal or neuroendocrine cell clusters *ASCL1* was used, for trunk and ductal clusters *HES1*, *SAT1* were used, and for tip and acinar clusters *CTRB1*, *GP2*, *RBPJL* were used. Endocrine progenitors were identified based on the expression of progenitor marker genes *SOX4* and *NEUROG3*, precursors using marker gene *FEV* and PAX4 (β-cell lineage) and ARX (α-cell lineage) amongst others.

### 4.14 Inference of β-cell dynamics using RNA velocity

To infer cellular dynamics in β-cells, RNA velocities were estimated for each human donor with a steady-state model as initially proposed by [60] and adopted and extended by [59] and in the scVelo python package (v0.2.2, https://github.com/theislab/scvelo). Splicing information of reads (spliced/unspliced) was extracted from the bam-files using the velocyto pipeline (http://velocyto.org). The resulting loompy file was then read into an AnnData object for downstream analysis with scVelo and Scanpy. To estimate velocities and infer cellular transitions the following steps were performed as recommended. First, genes were filtered with shared spliced and unspliced expression in less than 10 cells, the spliced and unspliced count layers were normalized to the initial total count per cell and log transformed (log (count+1)), and top 4000 variable genes were selected. Next, first- and second-order moments were calculated for each cell across its nearest neighbors of a kNN in principal components space (number of neighbors = 30, number of PCs = 30). Then velocities were estimated by fitting a steady-state model of transcription for each gene. Finally, a velocity graph was computed from the cosine similarities between the cell state change predicted by the velocity vector and possible cell transitions in the kNN. To compute the graph only genes with a likelihood >0.1 were considered. Using this graph the estimated velocities were then projected to the original UMAP space. To identify enriched velocity genes in mature and immature cells a differential expression test on velocities was applied comparing the velocity of one to all other clusters (Welch t-test with overestimated variance, tl. rank_velocity_genes). The velocity pseudotime was computed based on the directed velocity graph as implemented in scVelo (tl.velocity_pseudotime). The velocity pseudotime is a directed random-walk based distance measure between cells.

### 4.15 Cross-species mapping of α- and β-cell states

Mouse and pig α- and β-cells were mapped separately onto the human α- and β-cell reference states using the Scanpy ingest functionality (tl.ingest). Briefly, genes were subset to mappable genes and cells were scored for activation of the identified human gene sets. The gene set score matrix was scaled to standard variation (pp.scale). A single-cell manifold was then computed for human cells in gene set space applying the UMAP algorithm on the calculated kNN in PC space. Mouse and pig cells were mapped to the human reference through projecting to the PC space of the human cells. To map the single-cell embedding the UMAP package is used. Cell type labels are mapped using a kNN classifier.

Additional publicly available mouse data to confirm the cross species mapping were downloaded from GEO with accession number GSE162512 [67] and an AnnData object was generated. Cells with less than 200 total counts or 200 total genes expressed were filtered. Genes expressed in less than 10 cells were excluded. Raw counts of each cell were normalized by total counts of that cell not considering highly expressed genes for the total count normalization factor of a cell (pp.normalize_total with *exclude_highly_expressed* = True) and log-transformed (log (count+1)). Single-cell manifold generation, clustering and cluster annotation were performed as described above for the data of this study using top 2000 highly variable genes, 50 top principal components, a neighborhood size of 15 and known marker genes.

### 4.16 Mapping of Patch-Seq data to α- and β-cell states

Raw count matrices and metadata files including cell type annotations of Patch-Seq data from α- and β-cells generated by [4] were downloaded from https://github.com/jcamunas/patchseq/tree/master/data. An AnnData object was generated from the text-files for downstream analysis. Genes expressed in less than 5 cells or with less than 10 total counts were excluded. Raw counts of each cell were normalized by total counts of that cell. Counts were then log-transformed (log (count+1)). Data was subset to α- and β-cells using the provided cell type labels and mapped to our human reference states as described above for the cross-species mapping. Genes in gene sets were subset to 15′864 overlapping genes between the two studies before scoring. The data was then subset to patch-clamped cells from healthy donors. Cell states with <3 cells were excluded.

### 4.17 Mapping of 9 publicly available datasets to β-cell states

Raw count matrices and metadata of publicly available single-cell RNAseq datasets of pancreatic islets of healthy human donors were downloaded from GEO from accession numbers GSE114297 [9], GSE84133 [13], GSE86469 [55], GSE85241 [14], GSE81547 [25], GSE183568 [56], GSE101207 [58], and the cellxgene data portal (https://cellxgene.cziscience.com/collections/51544e44-293b-4c2b-8c26-560678423380) [57]. An AnnData object was generated for downstream analysis. Cells with less than 200 total counts or genes expressed were filtered. Genes expressed in less than 10 cells were excluded. Raw counts of each cell were normalized by total counts of that cell not considering highly expressed genes for the total count normalization factor of a cell (pp.normalize_total with *exclude_highly_expressed* = True) and log-transformed (log (count+1)). Additionally, the processed count matrix was downloaded from ArrayExpress (EBI) with accession number E-MTAB-5061 [23], an AnnData object was generated and counts were log-transformed (log (count+1)).

Single-cell manifold generation, clustering and cluster annotation were performed as described above for the data of this study using top 2000 highly variable genes, 50 top principal components, a neighborhood size of 15 and known marker genes. For GSE81547 [25] and GSE101207 [58] data of individual donors was integrated before computing the UMAP and clusters using the BBKNN alignment method [84]. For datasets from E-MTAB-5061 [23], GSE84133 [13,57] original cell type labels were kept.

The datasets of each study were then subset to α- and β-cells using the cell type labels and mapped to our human reference states as described above for the cross-species mapping. Genes in gene sets were subset to genes overlapping with this study before scoring.

### 4.18 Data and code availability

Annotated single-cell data can be explored and queried in the cellxgene data portal (https://cellxgene.cziscience.com/collections/0a77d4c0-d5d0-40f0-aa1a-5e1429bcbd7e) and were added to the sfaira data zoo [27]. Pig data was mapped and subset to human genes in the cellxgene portal. Raw data and count matrices of scRNA-seq data are available on GEO (accession number: GSE198623). Custom python scripts written for performing scRNA-seq analysis are available as jupyter notebooks in a github repository (https://github.com/theislab/2022_Tritschler_pancreas_cross_species). Python package versions that may affect numerical results as well as specific parameters and threshold values for all analyses are indicated in the scripts.

## CRediT author contributions

S.T.: Conceptualization, Methodology, Software, Data curation and analysis, Visualization, Writing- Original draft; M.T.: Software, Data curation and analysis, Writing- Reviewing and Editing, A.B.: Investigation, B.L.: Resources; J.S.: Investigation, Resources; U.S.: Investigation, Resources; E.K.: Investigation; E.W.: Supervision; H.L.: Conceptualization, Supervision, Resources, Funding acquisition, Writing- Reviewing and Editing; F.J.T.: Conceptualization, Supervision, Resources, Funding acquisition, Writing- Reviewing and Editing

## Data Availability

Data and source code were made publicly available on GEO (accession number: GSE198623) and in a github repository (https://github.com/theislab/2022_Tritschler_pancreas_cross_species). Data can be explored and queried in the cellxgene data portal (https://cellxgene.cziscience.com/collections/0a77d4c0-d5d0-40f0-aa1a-5e1429bcbd7e).

## Appendix A Supplementary data

Supplementary_Table_1 Cell type composition per samples. Supplementary_Table_1

Supplementary_Table_2 Expression and conservation of mappable genes in endocrine cell types. Supplementary_Table_2

Supplementary_Table_3 Enriched marker genes of endocrine cell types and their conservation. Supplementary_Table_3

Supplementary_Table_4 Human α- and β-cell gene sets. Supplementary_Table_4

Supplementary_Table_5 Differential velocity genes with high expression in human mature and immature β-cell states. Supplementary_Table_5

Suppl_Figures_final **Supplementary Figure 1 Conservation of gene expression in scRNA-seq data of human, mouse and pig islet cells.** A) Metadata of the 5 human donors. ID indicates donor ID for ADI IsletCore (see Material and Methods). B) Quality control metrics of scRNA-seq data. C) Scatter plot of the top two principal components. Cells are colored by species. D) Summary of conservation of human gene expression in pig and mouse in endocrine cell types. A gene is considered expressed if detected in >5% of the cells of the cell type. E) Expression of selected genes in human, pig and mouse endocrine cell types exemplifying conservation, “gain” and “loss” of expression shown in C. Color intensity indicates mean expression in a cluster, dot size indicates the proportion of cells in a cluster expressing the gene. F) Comparison of conserved β-cell genes to β-cell core genes derived from human and mouse bulk β-cell transcriptomes [36]. Left: Venn Diagram showing the overlap of reported β-cell core genes (9’474) and our list of mappable genes (11’665). Right: Barplot indicating conservation of 8105 overlapping β-cell core genes between human and mouse β-cells. A gene is considered expressed if detected in >5% of the cells of the cell type. G-I) Pairwise correlation of TF expression patterns between species for each cell type. Pearson correlation is computed on a subset of TF as indicated using the harmonic average of mean expression and fraction of cells expressing a gene in a group across all cell types (Material and Methods). Pearson correlation coefficient is indicated. G) Cell-type enriched marker TFs conserved across species as shown in Figure 1G. H) All TFs enriched in human cell-types. I) All TFs with conserved expression across species. **Supplementary Figure 2 Transcriptional profiling of human β-cell states.** A) Cell scores indicating hallmark pathway activation in β-cell clusters. Top 5 enriched hallmarks are shown per cluster. Scaled scores per pathway are shown. B) Expression of β-cell disallowed genes in β-cell clusters. Color intensity indicates mean expression in a cluster, dot size indicates the proportion of cells in a cluster expressing the gene. Expression is scaled per gene. C) Variance of non-scaled gene set scores across all β-cells indicating magnitude of activation level differences across clusters. D-F) Comparison of the transcriptional profile of the identified MHC/autoantigen β-cell cluster to β-cells from T1D patients [42]. D) UMAP plot of β-cells from healthy and T1D patients. T1D score indicates increased expression of T1D-associated genes in the MHC/autoantigen cluster. The T1D score is computed from the top differentially expressed genes between β-cells of T1D patients and healthy individuals. E) Expression of MHC genes in healthy and T1D β-cells. F) Gene sets increased in MHC/autoantigen cluster are also increased in T1D β-cells. G) UMAP plot of endocrine cells colored by MHC/autoantigen gene set (G9) scores. Circles highlight clusters with high activation scores in α-, β- and δ-cells. H) Expression of RNA polymerase II and general transcription and translation factors expressed in >200 β-cells. Color intensity indicates mean expression in a cluster, dot size indicates the proportion of cells in a cluster expressing the gene. Expression is scaled per gene. I) UMAP plot of human β-cells colored by data quality metrics. *Top*: Percentage of counts from mitochondria-encoded RNA, *middle*: total number of counts per cell, *bottom*: total number of genes per cell. J) Cells scores indicating stress pathway activation in β-cell clusters. Scores were computed based on the expression of genes in the corresponding GO pathways (see Methods). K) Expression of transcription, signaling and growth factors in β-cell clusters. Genes were described to be significantly downregulated by glucocorticoid signaling in human islets [45]. Color intensity indicates mean expression in a cluster, dot size indicates the proportion of cells in a cluster expressing the gene. Expression is scaled per gene. L,M) Expression of ion channels (L) and selected components of cAMP signaling pathway (M) expressed in >200 β-cells. Color intensity indicates mean expression in a cluster, dot size indicates the proportion of cells in a cluster expressing the gene. Expression is scaled per gene. N,O) Excitability of β-cell states measured in single-cell Patch-Seq data [4]. State labels were mapped in the β-cell gene set representation using the Scanpy ingest functionality. N) β-cell gene set activation in Patch-Seq cells. Scaled mean scores for each gene set per β-cell state are shown. O) Boxplots showing the distribution of different electrophysiological measurements per β-cell state (*top left*). Line indicates the median, values are FDR of differential test against mature state. Extreme values above 97% or below 3% - quantiles were excluded. Data were analyzed by a Mann-Withney-U test and Benjamini-Hochberg correction for multiple testing per state comparison. *Top left:* Barplot showing β-cell state composition and total number of cells per state. Error bar indicating donor variation. **Supplementary Figure 3 Cross-study mapping of β-cell states.** A-C) β-cell states across 9 studies and 54 donors. A) Reference UMAP showing β-cells in gene set representation, where each cell is represented by an activation score of the corresponding cell gene sets. B) Mapping of β-cells from publicly available studies to the reference UMAP in A. Cells were mapped through projecting on the reference gene set representation. Embedding and labels are mapped using the Scanpy ingest functionality (see Methods). The barplot indicates the frequencies of mapped clusters. Number of donors and median numbers of cells and genes per donor are indicated. C) β-cell gene set activation in mapped β-cell states per study. D) Barplot showing fraction of β-cell states in male and female donors of all studies. N.a. indicates donor for which sex information was not available. E) Scatterplots showing linear relationship between fraction of cells per β-cell cluster and age. Line shows linear regression fit, shaded area shows the 95% confidence interval for the regression. Pearson correlation coefficient (r) and p-value (p) testing for non-correlation are indicated. **Supplementary Figure 4 RNA velocity analysis in β-cell across human donors.** A) Cellular dynamics in β-cells resolved by donor. Cell transitions are inferred from estimated RNA velocities and the direction of inferred movement plotted as streamlines on the UMAP. Colors indicate β-cell clusters. B) Dotplots showing mean velocities per β-cell cluster resolved by donor. Selected known genes involved in β-cell maturation and potential novel genes important for maturation are shown. C) Inferred high or low velocity clusters of mature β-cells. *Top:* UMAP indicating clustering into high or low velocity cells. *Bottom:* Expression of genes previously described to separate CD9+ and CD9- β-cells in high and low velocity mature β-cells. Color intensity indicates mean expression in a cluster, dot size indicates the proportion of cells in a cluster expressing the gene. Expression is scaled per gene. **Supplementary Figure 5 Maturation factor expression in human fetal β-cell development of publicly available datasets.** A-H) Comparison of identified immature β-cell cluster in adult islets to fetal β-cell development. A-D) Single cell sequencing data of fetal pancreata from [64]. E-H) Single cell sequencing data of fetal pancreata from [65]. A, E) UMAP plot of endocrine lineage cells isolated from fetal human pancreases. Colors indicate clusters of differentiation states from Ngn3+ endocrine progenitors (EP) or Fev+ precursors, respectively, to immature endocrine cells. B, F) Expression of known β-cell identity and maturity genes. C, G) Expression of genes driving inferred β-cell maturation dynamics (see Figure 3C). Color intensity indicates mean expression in a cluster, dot size indicates the proportion of cells in a cluster expressing the gene. D, H) Activation of adult β-cell gene sets (see Figure 2E) in fetal progenitor/precursor and β-cell clusters. Scaled mean scores for each gene set per β-cell cluster are shown. **Supplementary Figure 6 Transcriptional profiling of human α-cell states.** A, B) Comparison of adult α-cell states to fetal α-cell development from [64] (Cao et al 2022) and [65] (Yu et al 2021), see also Figure S5A. A) Activation of adult α-cell gene sets (see Figure 4F) in fetal precursor and α-cell clusters. Scaled mean scores for each gene set per α-cell cluster are shown. B) Expression of α-cell identity and maturation factors as well as developmental factors and genes of the TGFβ signaling pathway. C) Silhouette scores [85] as a proxy of cluster similarity and homogeneity. Violinplots show distribution of silhouette scores per β-cell (left) and α-cell (right) cluster. Silhouette scores were computed on the 50 top principal components using euclidean distance. D-G) Excitability of α-cell states measured in single-cell Patch-Seq data [4]. State labels were mapped in the α-cell gene set representation using the Scanpy ingest functionality D) Barplot showing α-cell state composition and total number of cells per state. Error bar indicating donor variation. E) α-cell gene set activation in Patch-Seq cells. Scaled mean scores for each gene set per α-cell cluster are shown. F) Expression of α-cell identity and maturation factors as well as genes involved in pathways describing immature α-cells. G) Boxplot showing distribution of different electrophysiological measurements per α-cell state. Line indicates the median, values are FDR of differential test against mature state. Data were analyzed by a Mann-Withney-U test and Benjamini-Hochberg correction for multiple testing per state comparison. **Supplementary Figure 7 Conservation of human α- and β-cell state signatures in pig and mouse.** A-D) Conservation of the human β-cell states A) β-cell gene set activation scores for β-cell clusters across species. B) Pearson correlation matrix of gene expression of β-cell clusters across species. β-cell clusters are grouped by hierarchical clustering. C,D) Expression of β-cell identity and maturity markers (C) and genes associated with a stress-response (D) in β-cell clusters across species. Color intensity indicates mean expression in a cluster, dot size indicates the proportion of cells in a cluster expressing the gene. Expression is scaled per gene. E-I) Conservation of the human α-cell states. E) α-cell gene set activation scores for α-cell clusters across species. F) Pearson correlation matrix of gene expression of α-cell clusters across species. α-cell clusters are grouped by hierarchical clustering. G-H) Expression of α-cell identity markers (G), genes describing immature human α-cells (H) and stress-associated genes (I) in α-cell clusters across species. Color intensity indicates mean expression in a cluster, dot size indicates the proportion of cells in a cluster expressing the gene. Expression is scaled per gene. J) Barplot indicating conservation of gene expression in mature α- (left) and β- (right) cells from pig and mouse. Conservation of mappable genes within α- or β-cell maturity gene sets is shown. Genes are considered expressed if detected in >5% of mature cells. K) Expression of identified β-cell maturation markers in β-cell clusters across species. Color intensity indicates mean expression in a cluster, dot size indicates the proportion of cells in a cluster expressing the gene. Expression is scaled per gene. L) Expression of selected genes in β-cells of scRNA-seq data from vehicle and STZ-treated diabetic mice [11]. Color intensity indicates mean expression in a cluster, dot size indicates the proportion of cells in a cluster expressing the gene. Expression is scaled per gene. **Supplementary Figure 8 Cross-species mapping of human α- and β-cell states using a publicly available mouse dataset.** A-D) Conservation of the human α- and β-cell states in mouse cells of a publicly available mouse dataset [67] A,C) Mapping of mouse α- (A) and β-cells (C) to the human reference UMAP. Cells were mapped through projecting on the reference gene set representation. Embedding and labels are mapped using the Scanpy ingest functionality (see Methods). The barplot indicates the frequencies of mapped clusters. Number of mice, total and median numbers of cells and genes per mouse are indicated. B,D) α- (B) and β-cell (D) gene set activation in mapped α- and β-cell states in [67]. Suppl_Figures_final
