# Supplementary material for: A transcriptional cross species map of pancreatic islet cells
Source: Mol Metab. 2022 Sep 13;66:101595. doi: 10.1016/j.molmet.2022.101595 (PMC9526148; doi:10.1016/j.molmet.2022.101595)
Supplement: Suppl_Figures_final — Supplementary Figure 1 Conservation of gene expression in scRNA-seq data of human, mouse and pig islet cells. A) Metadata of the 5 human donors. ID indicates donor ID for ADI IsletCore (see Material and Methods). B) Quality control metrics of scRNA-seq data. C) Scatter plot of the top two principal components. Cells are colored by species. D) Summary of conservation of human gene expression in pig and mouse in endocrine cell types. A gene is considered expressed if detected in >5% of the cells of the cell type. E) Expression of selected genes in human, pig and mouse endocrine cell types exemplifying conservation, “gain” and “loss” of expression shown in C. Color intensity indicates mean expression in a cluster, dot size indicates the proportion of cells in a cluster expressing the gene. F) Comparison of conserved β-cell genes to β-cell core genes derived from human and mouse bulk β-cell transcriptomes [36]. Left: Venn Diagram showing the overlap of reported β-cell core genes (9’474) and our list of mappable genes (11’665). Right: Barplot indicating conservation of 8105 overlapping β-cell core genes between human and mouse β-cells. A gene is considered expressed if detected in >5% of the cells of the cell type. G-I) Pairwise correlation of TF expression patterns between species for each cell type. Pearson correlation is computed on a subset of TF as indicated using the harmonic average of mean expression and fraction of cells expressing a gene in a group across all cell types (Material and Methods). Pearson correlation coefficient is indicated. G) Cell-type enriched marker TFs conserved across species as shown in Figure 1G. H) All TFs enriched in human cell-types. I) All TFs with conserved expression across species. Supplementary Figure 2 Transcriptional profiling of human β-cell states. A) Cell scores indicating hallmark pathway activation in β-cell clusters. Top 5 enriched hallmarks are shown per cluster. Scaled scores per pathway are shown. B) Expression of β-cell [file mmc6.pdf]

Supplementary Figure 1 Conservation of gene expression in scRNA-seq data of human, mouse and pig islet cells

A

ID	sex	age	BMI	HbAC1 %
R229	female	22	23,0	5,3
R239	female	24	22,0	5,5
R237	male	61	19,6	5,9
R245	male	63	22,3	5,6
R266	female	74	29,2	6,0

B

sample	# cells	median #counts/cell	median #genes/cell
human	26474	15277	3159
pig	22056	8831	1836
mouse	7011	16927	2423

C

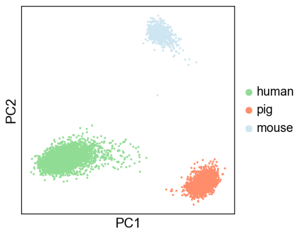

D

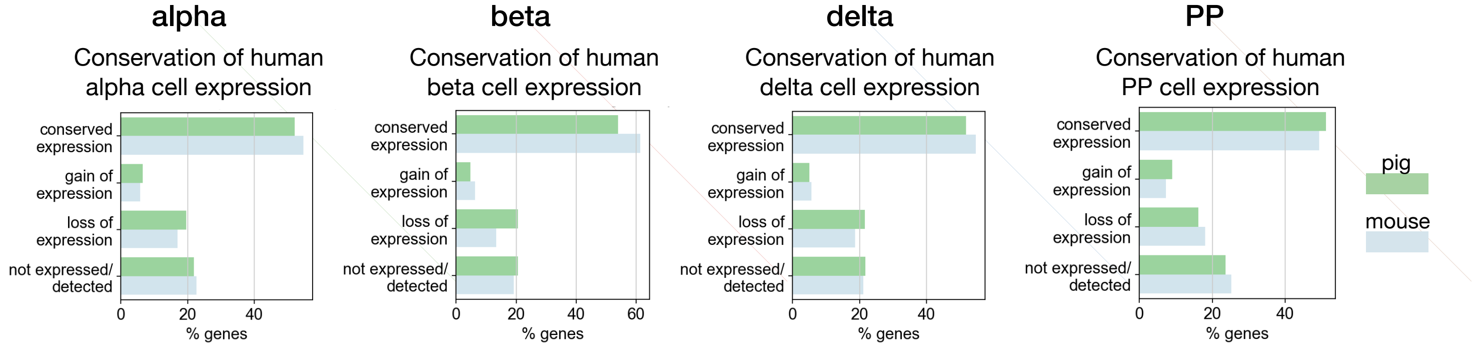

E

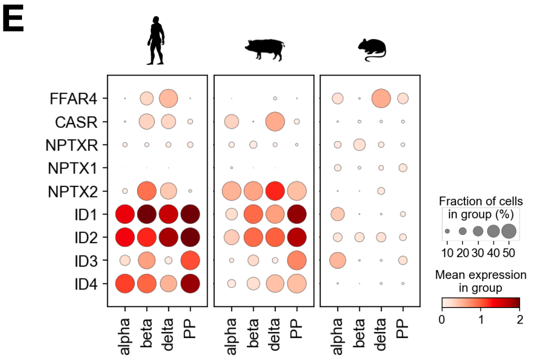

F

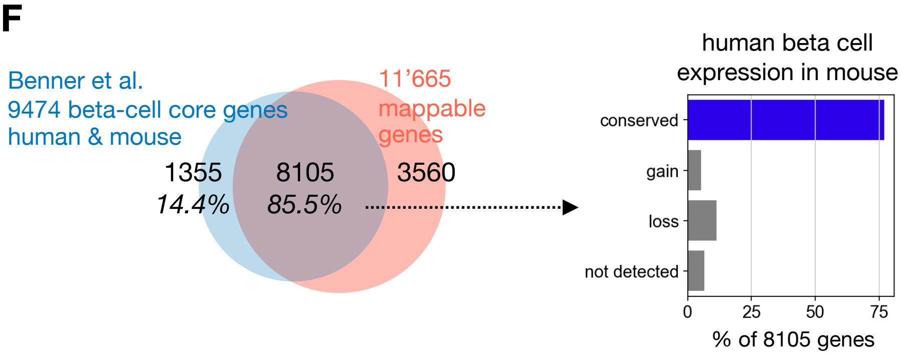

G **H** **I**

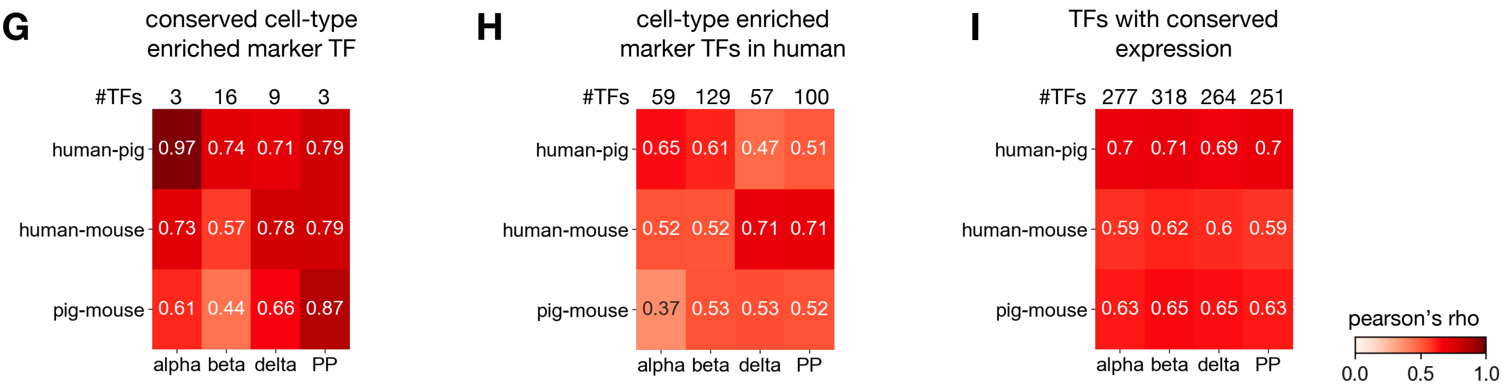

Supplementary Figure 1 Conservation of gene expression in scRNA-seq data of human, mouse and pig islet cells

A) Metadata of the 5 human donors. ID indicates donor ID for ADI IsletCore (see Material & Methods).

B) Quality control metrics of scRNA-seq data.

C) Scatter plot of the top two principal components. Cells are colored by species.

D) Summary of conservation of human gene expression in pig and mouse in endocrine cell types. A gene is considered expressed if detected in >5% of the cells of the cell type.

E) Expression of selected genes in human, pig and mouse endocrine cell types exemplifying conservation, “gain” and “loss” of expression shown in C. Color intensity indicates mean expression in a cluster, dot size indicates the proportion of cells in a cluster expressing the gene.

F) Comparison of conserved β-cell genes to β-cell core genes derived from human and mouse bulk β-cell transcriptomes [36]. Left: Venn Diagram showing the overlap of reported β-cell core genes (9’474) and our list of mappable genes (11’665). Right: Barplot indicating conservation of 8105 overlapping β-cell core genes between human and mouse β-cells. A gene is considered expressed if detected in >5% of the cells of the cell type.

G-I) Pairwise correlation of TF expression patterns between species for each cell type. Pearson correlation is computed on a subset of TF as indicated using the harmonic average of mean expression and fraction of cells expressing a gene in a group across all cell types (Material & Methods). Pearson correlation coefficient is indicated. G) Cell-type enriched marker TFs conserved across species as shown in Figure 1G. H) All TFs enriched in human cell-types. I) All TFs with conserved expression across species.

Supplementary Figure 2 Transcriptional profiling of human β-cell states

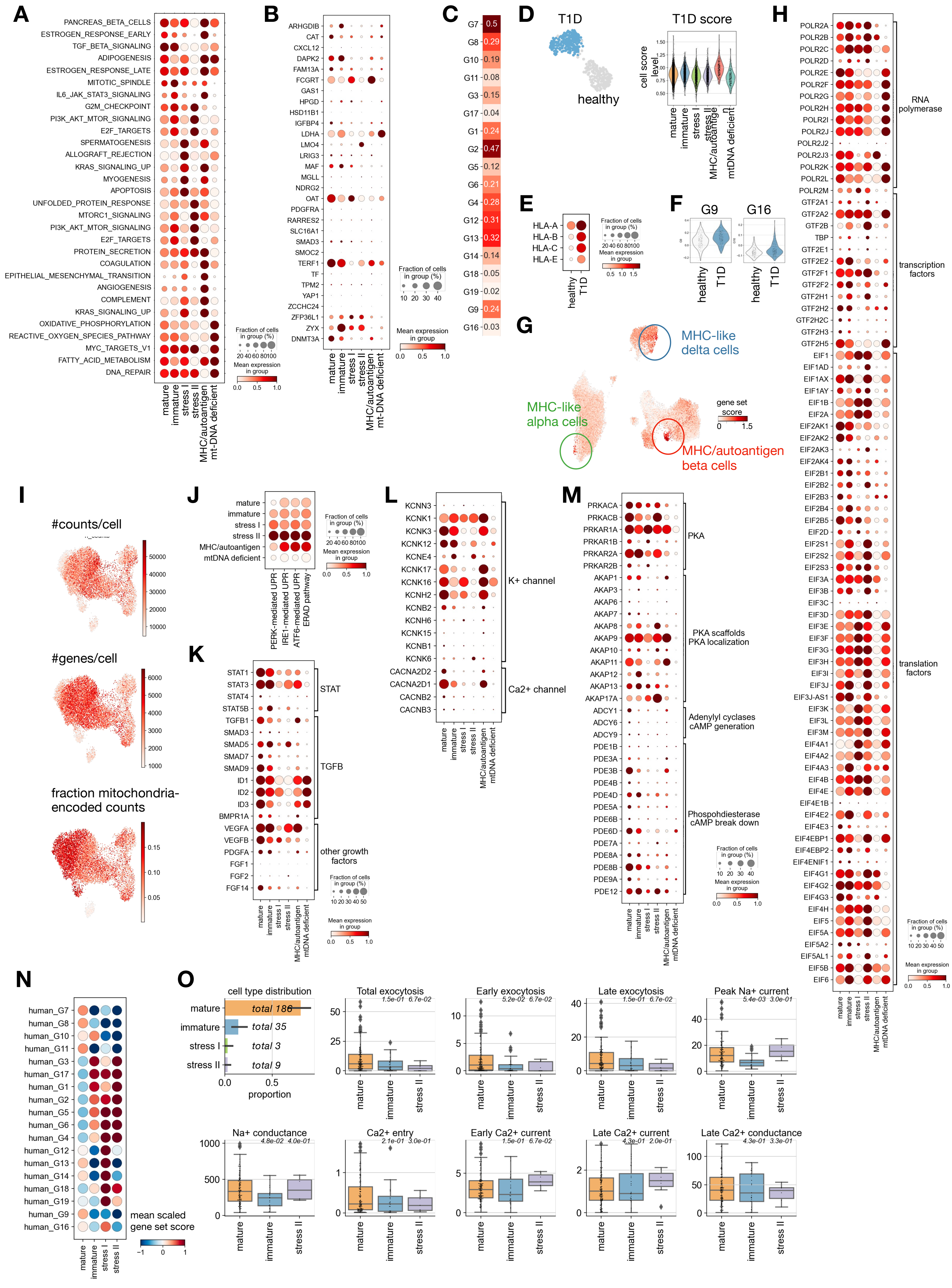

Supplementary Figure 2 Transcriptional profiling of human β-cell states

A) Cell scores indicating hallmark pathway activation in β-cell clusters. Top 5 enriched hallmarks are shown per cluster. Scaled scores per pathway are shown.

B) Expression of β-cell disallowed genes in β-cell clusters. Color intensity indicates mean expression in a cluster, dot size indicates the proportion of cells in a cluster expressing the gene. Expression is scaled per gene.

C) Variance of non-scaled gene set scores across all β-cells indicating magnitude of activation level differences across clusters.

D-F) Comparison of the transcriptional profile of the identified MHC/autoantigen β-cell cluster to β-cells from T1D patients [42]. D) UMAP plot of β-cells from healthy and T1D patients. T1D score indicates increased expression of T1D-associated genes in the MHC/autoantigen cluster. The T1D score is computed from the top differentially expressed genes between β-cells of T1D patients and healthy individuals. E) Expression of MHC genes in healthy and T1D β-cells. F) Gene sets increased in MHC/autoantigen cluster are also increased in T1D β-cells.

G) UMAP plot of endocrine cells colored by MHC/autoantigen gene set (G9) scores. Circles highlight clusters with high activation scores in α-, β- and δ-cells.

H) Expression of RNA polymerase II and general transcription and translation factors expressed in >200 β-cells. Color intensity indicates mean expression in a cluster, dot size indicates the proportion of cells in a cluster expressing the gene. Expression is scaled per gene.

I) UMAP plot of human β-cells colored by data quality metrics. Top: Percentage of counts from mitochondria-encoded RNA, middle: total number of counts per cell, bottom: total number of genes per cell.

J) Cells scores indicating stress pathway activation in β-cell clusters. Scores were computed based on the expression of genes in the corresponding GO pathways (see Methods).

K) Expression of transcription, signaling and growth factors in β-cell clusters. Genes were described to be significantly downregulated by glucocorticoid signaling in human islets [45]. Color intensity indicates mean expression in a cluster, dot size indicates the proportion of cells in a cluster expressing the gene. Expression is scaled per gene.

L,M) Expression of ion channels (L) and selected components of cAMP signaling pathway (M) expressed in >200 β-cells. Color intensity indicates mean expression in a cluster, dot size indicates the proportion of cells in a cluster expressing the gene. Expression is scaled per gene.

N,O) Excitability of β-cell states measured in single-cell Patch-Seq data [4]. State labels were mapped in the β-cell gene set representation using the Scanpy ingest functionality. N) β-cell gene set activation in Patch-Seq cells. Scaled mean scores for each gene set per β-cell state are shown. O) Boxplots showing the distribution of different electrophysiological measurements per β-cell state (top left). Line indicates the median, values are FDR of differential test against mature state. Extreme values above 97% or below 3% - quantiles were excluded. Data were analyzed by a Mann-Withney-U test and Benjamini-Hochberg correction for multiple testing per state comparison. Top left: Barplot showing β-cell state composition and total number of cells per state. Error bar indicating donor variation.

Supplementary Figure 3 Cross-study mapping of β-cell states

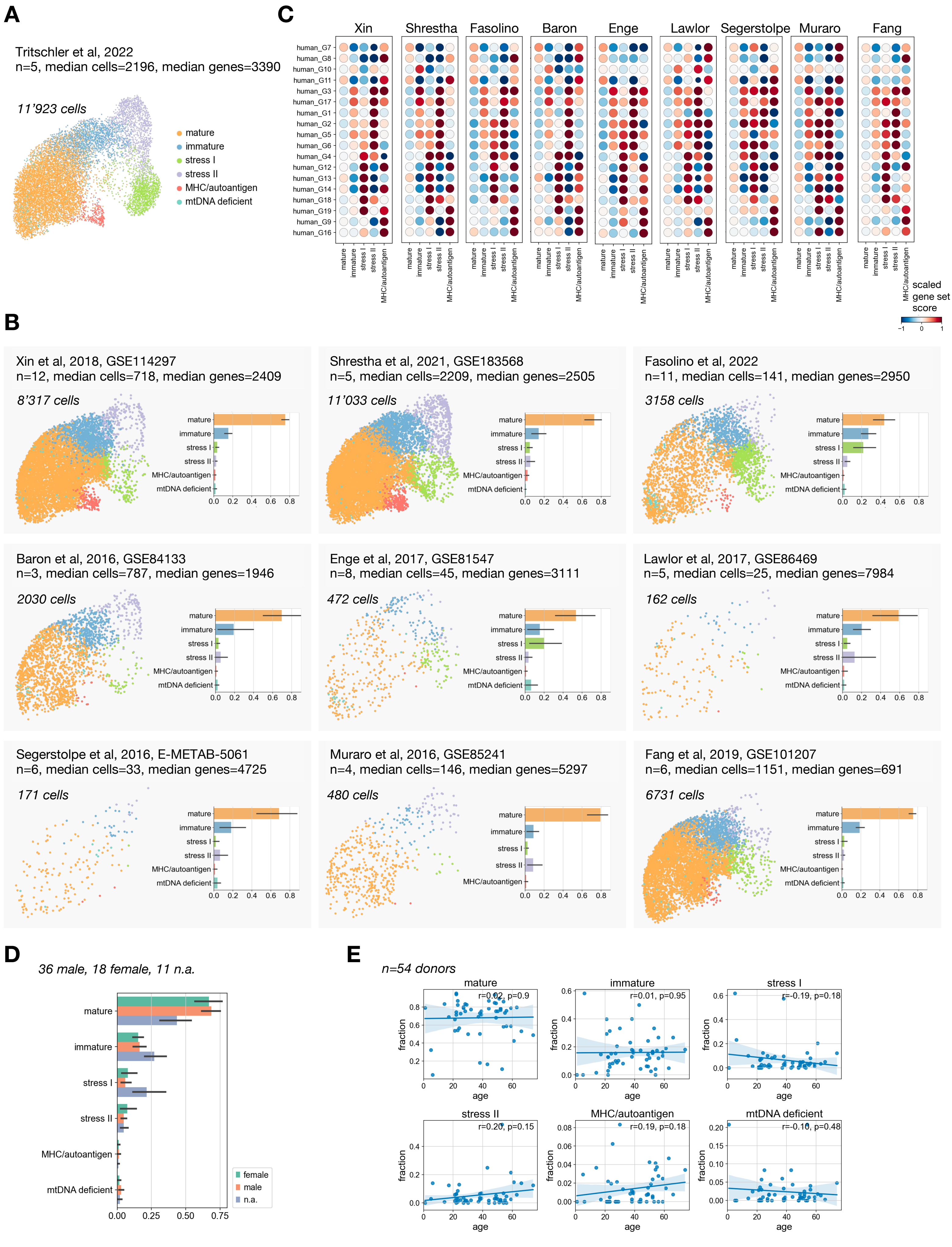

Supplementary Figure 3 Cross-study mapping of β-cell states

A-C) β-cell states across 9 studies and 54 donors. A) Reference UMAP showing β-cells in gene set representation, where each cell is represented by an activation score of the corresponding cell gene sets. B) Mapping of β-cells from publicly available studies to the reference UMAP in A. Cells were mapped through projecting on the reference gene set representation. Embedding and labels are mapped using the Scanpy ingest functionality (see Methods). The barplot indicates the frequencies of mapped clusters. Number of donors and median numbers of cells and genes per donor are indicated.

C) β-cell gene set activation in mapped β-cell states per study.

D) Barplot showing fraction of β-cell states in male and female donors of all studies. N.a. indicates donor for which sex information was not available.

E) Scatterplots showing linear relationship between fraction of cells per β-cell cluster and age. Line shows linear regression fit, shaded area shows the 95% confidence interval for the regression. Pearson correlation coefficient (r) and p-value (p) testing for non-correlation are indicated.

Supplementary Figure 4 RNA velocity analysis in β-cell across human donors

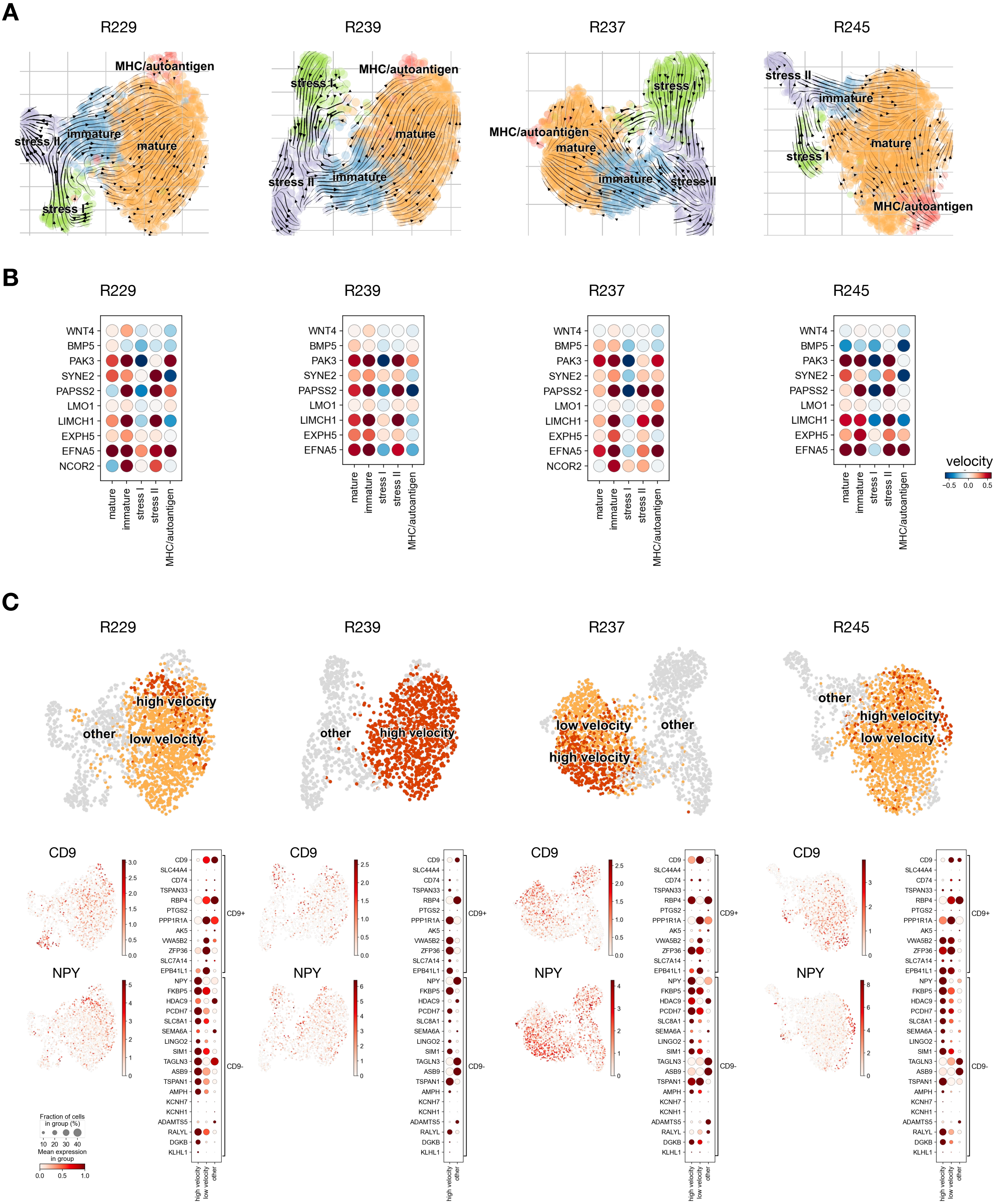

Supplementary Figure 4 RNA velocity analysis in β-cell across human donors

A) Cellular dynamics in β-cells resolved by donor. Cell transitions are inferred from estimated RNA velocities and the direction of inferred movement plotted as streamlines on the UMAP. Colors indicate β-cell clusters.

B) Dotplots showing mean velocities per β-cell cluster resolved by donor. Selected known genes involved in β-cell maturation and potential novel genes important for maturation are shown.

C) Inferred high or low velocity clusters of mature β-cells. *Top:* UMAP indicating clustering into high or low velocity cells. *Bottom:* Expression of genes previously described to separate CD9+ and CD9- β-cells in high and low velocity mature β-cells. Color intensity indicates mean expression in a cluster, dot size indicates the proportion of cells in a cluster expressing the gene. Expression is scaled per gene.

Supplementary Figure 5 Maturation factor expression in human fetal β-cell development in publicly available datasets

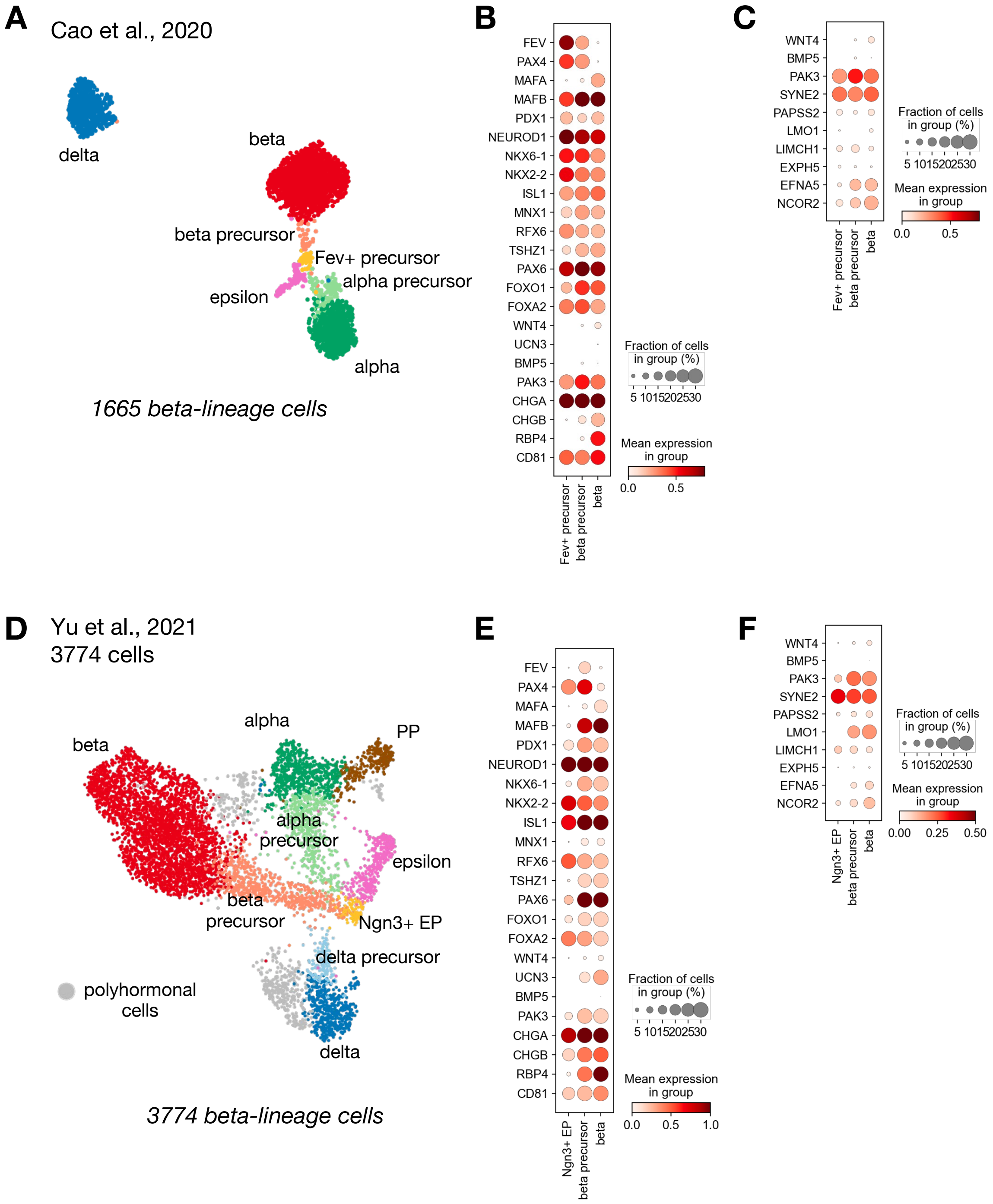

Supplementary Figure 5 Maturation factor expression in human fetal β-cell development of publicly available datasets

A-F) Comparison of identified immature β-cell cluster in adult islets to fetal β-cell development. A-C) Single cell sequencing data of fetal pancreata from [64]. D-F) Single cell sequencing data of fetal pancreata from [65]. A, D) UMAP plot of endocrine lineage cells isolated from fetal human pancreases. Colors indicate clusters of differentiation states from Ngn3+ endocrine progenitors (EP) or Fev+ precursors, respectively, to immature endocrine cells. B, E) Expression of known β-cell identity and maturity genes. C, F) Expression of genes driving inferred β-cell maturation dynamics (see Figure 3C). Color intensity indicates mean expression in a cluster, dot size indicates the proportion of cells in a cluster expressing the gene.

Supplementary Figure 6 Transcriptional profiling of human α-cell states

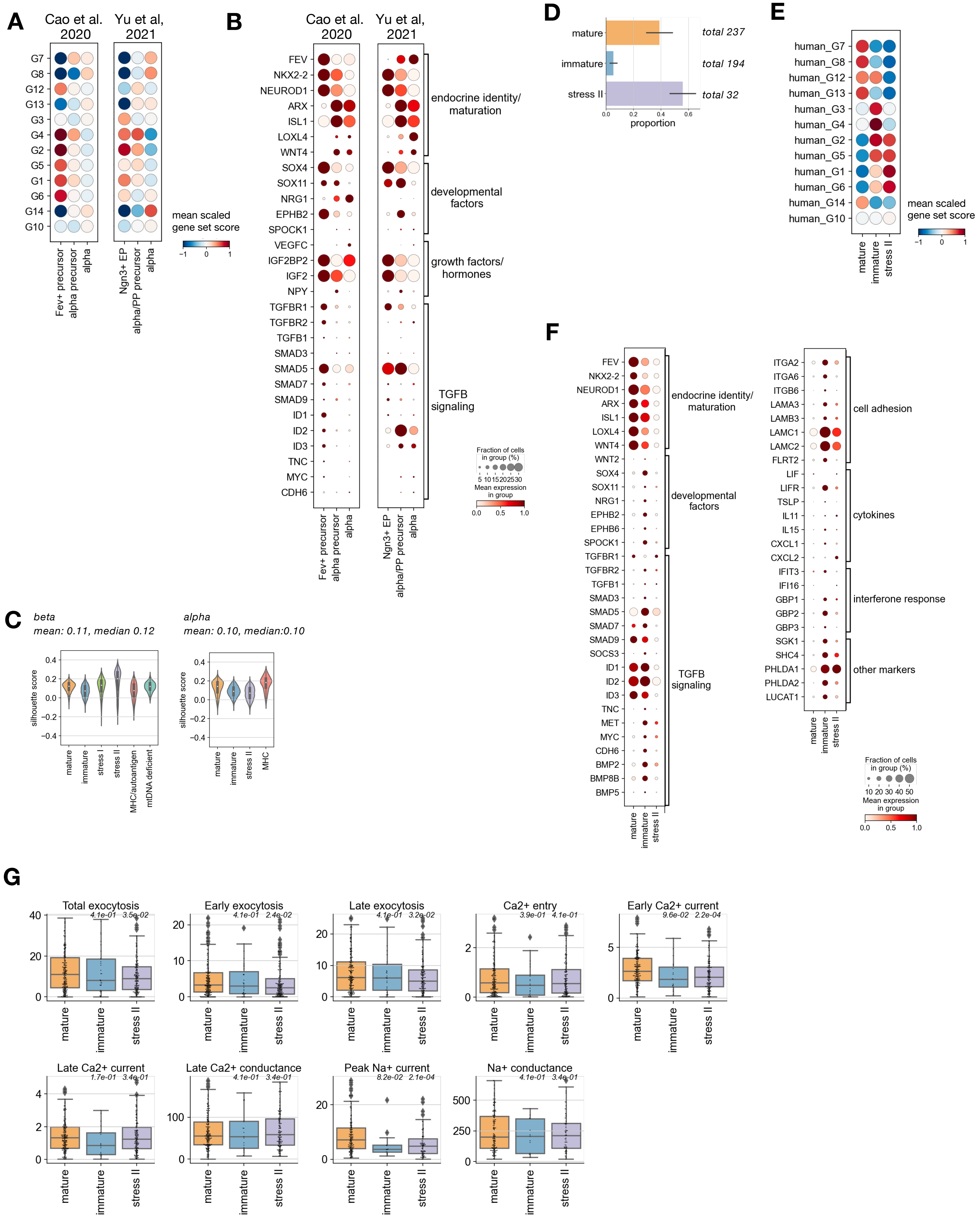

Supplementary Figure 6 Transcriptional profiling of human α-cell states

A, B) Comparison of adult α-cell states to fetal α-cell development from [64] (Cao et al 2022) and [65] (Yu et al 2021), see also Figure S5A. A) Activation of adult α-cell gene sets (see Figure 4F) in fetal precursor and α-cell clusters. Scaled mean scores for each gene set per α-cell cluster are shown. B) Expression of α-cell identity and maturation factors as well as developmental factors and genes of the TGFβ signaling pathway.

C) Silhouette scores [85] as a proxy of cluster similarity and homogeneity. Violinplots show distribution of silhouette scores per β-cell (left) and α-cell (right) cluster. Silhouette scores were computed on the 50 top principal components using euclidean distance.

D-G) Excitability of α-cell states measured in single-cell Patch-Seq data [4]. State labels were mapped in the α-cell gene set representation using the Scanpy ingest functionality D) Barplot showing α-cell state composition and total number of cells per state. Error bar indicating donor variation. E) α-cell gene set activation in Patch-Seq cells. Scaled mean scores for each gene set per α-cell cluster are shown. F) Expression of α-cell identity and maturation factors as well as genes involved in pathways describing immature α-cells. G) Boxplot showing distribution of different electrophysiological measurements per α-cell state. Line indicates the median, values are FDR of differential test against mature state. Data were analyzed by a Mann-Withney-U test and Benjamini-Hochberg correction for multiple testing per state comparison.

Supplementary Figure 7 Conservation of human α- and β-cell state signatures in pig and mouse

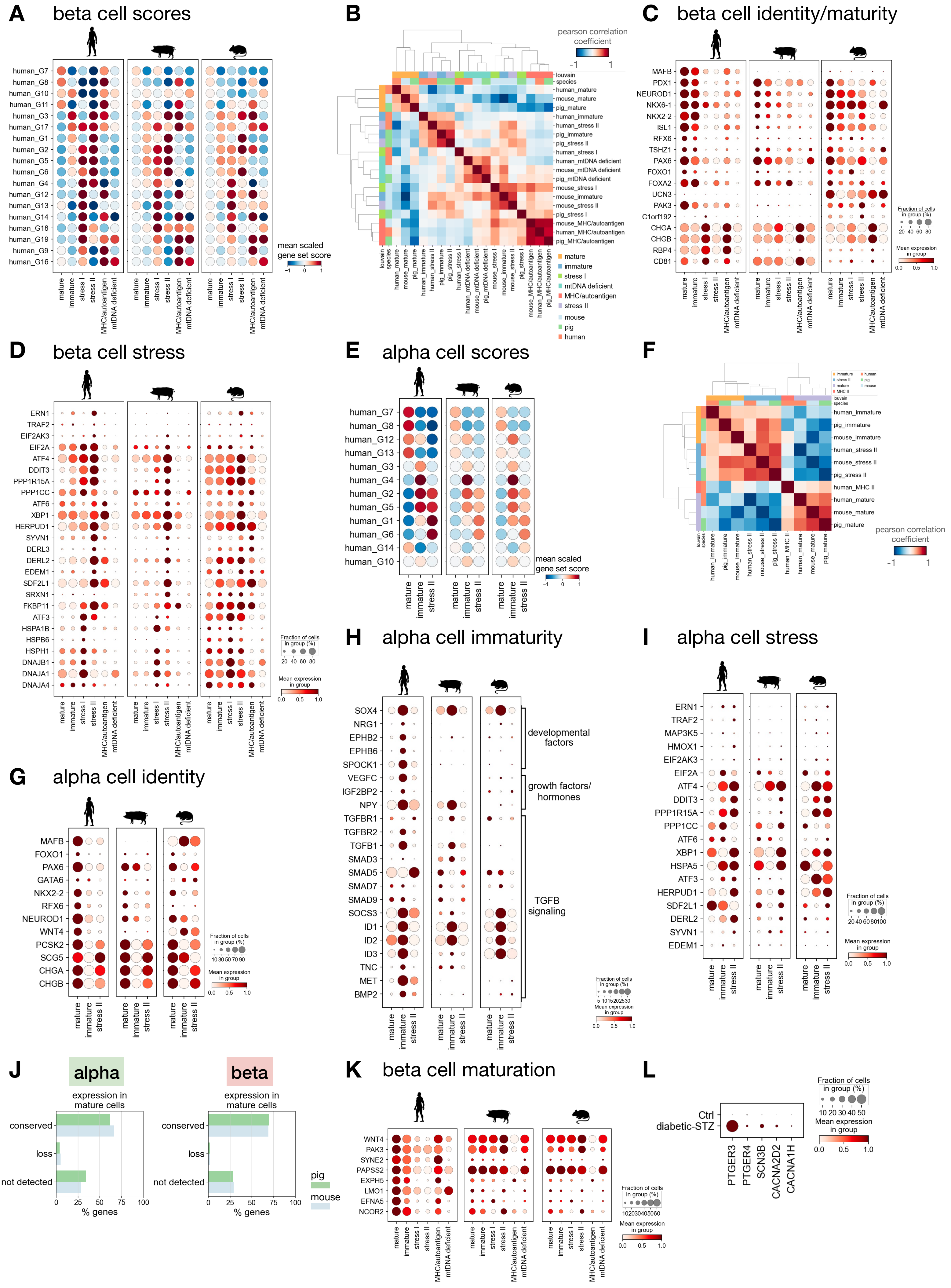

Supplementary Figure 7 Conservation of human α- and β-cell state signatures in pig and mouse

A-D) Conservation of the human β-cell states A) β-cell gene set activation scores for β-cell clusters across species. B) Pearson correlation matrix of gene expression of β-cell clusters across species. β-cell clusters are grouped by hierarchical clustering. C,D) Expression of β-cell identity and maturity markers (C) and genes associated with a stress-response (D) in β-cell clusters across species. Color intensity indicates mean expression in a cluster, dot size indicates the proportion of cells in a cluster expressing the gene. Expression is scaled per gene.

E-I) Conservation of the human α-cell states. E) α-cell gene set activation scores for α-cell clusters across species. F) Pearson correlation matrix of gene expression of α-cell clusters across species. α-cell clusters are grouped by hierarchical clustering. G-H) Expression of α-cell identity markers (G), genes describing immature human α-cells (H) and stress-associated genes (I) in α-cell clusters across species. Color intensity indicates mean expression in a cluster, dot size indicates the proportion of cells in a cluster expressing the gene. Expression is scaled per gene.

J) Barplot indicating conservation of gene expression in mature α- (left) and β- (right) cells from pig and mouse. Conservation of mappable genes within α- or β-cell maturity gene sets is shown. Genes are considered expressed if detected in >5% of mature cells.

K) Expression of identified β-cell maturation markers in β-cell clusters across species. Color intensity indicates mean expression in a cluster, dot size indicates the proportion of cells in a cluster expressing the gene. Expression is scaled per gene.

L) Expression of selected genes in β-cells of scRNA-seq data from vehicle and STZ-treated diabetic mice [11]. Color intensity indicates mean expression in a cluster, dot size indicates the proportion of cells in a cluster expressing the gene. Expression is scaled per gene.

Supplementary Figure 8 Cross-species mapping of human α- and β-cell states using a publicly available mouse dataset

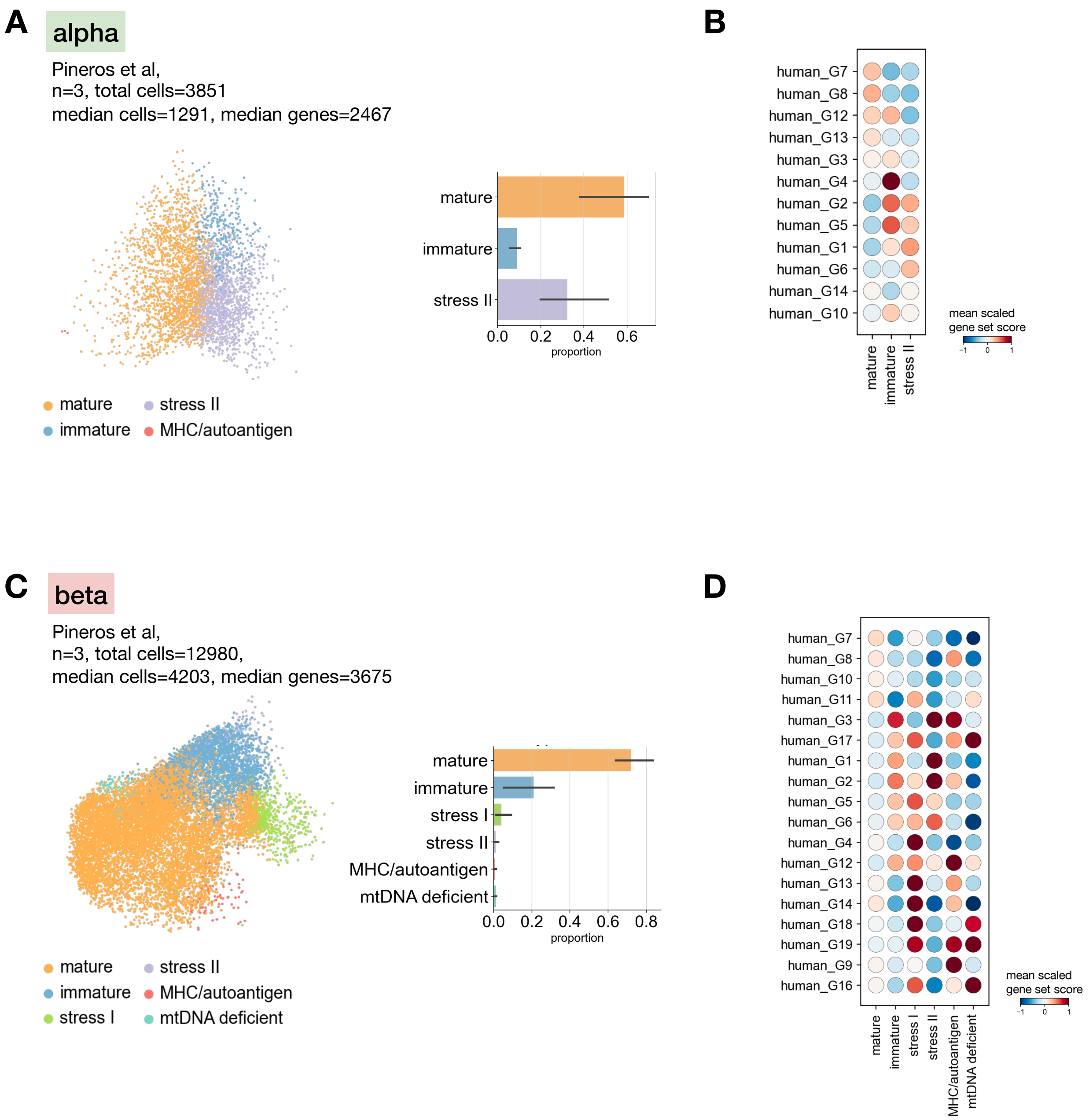

Supplementary Figure 8 Cross-species mapping of human α- and β-cell states using a publicly available mouse dataset

A-D) Conservation of the human α- and β-cell states in mouse cells of a publicly available mouse dataset [67] A,C) Mapping of mouse α- (A) and β-cells (C) to the human reference UMAP. Cells were mapped through projecting on the reference gene set representation. Embedding and labels are mapped using the Scanpy ingest functionality (see Methods). The barplot indicates the frequencies of mapped clusters. Number of mice, total and median numbers of cells and genes per mouse are indicated. B,D) α- (B) and β-cell (D) gene set activation in mapped α- and β-cell states in [67].
